# MSH2-MSH3 promotes DNA end resection during homologous recombination and blocks polymerase theta-mediated end-joining through interaction with SMARCAD1 and EXO1

**DOI:** 10.1093/nar/gkad308

**Published:** 2023-05-04

**Authors:** Jung-Min Oh, Yujin Kang, Jumi Park, Yubin Sung, Dayoung Kim, Yuri Seo, Eun A Lee, Jae Sun Ra, Enkhzul Amarsanaa, Young-Un Park, Seon Young Lee, Jung Me Hwang, Hongtae Kim, Orlando Schärer, Seung Woo Cho, Changwook Lee, Kei-ichi Takata, Ja Yil Lee, Kyungjae Myung

**Affiliations:** Center for Genomic Integrity, Institute for Basic Science (IBS), Ulsan 44919, Republic of Korea; Department of Oral Biochemistry, Dental and Life Science Institute, School of Dentistry, Pusan National University, Yangsan 50612, Republic of Korea; Department of Biological Sciences, Ulsan National Institute of Science and Technology, Ulsan44919, Republic of Korea; Department of Biological Sciences, Ulsan National Institute of Science and Technology, Ulsan44919, Republic of Korea; Center for Genomic Integrity, Institute for Basic Science (IBS), Ulsan 44919, Republic of Korea; Department of Biomedical Engineering, Ulsan National Institute of Science and Technology, Ulsan44919, Republic of Korea; Center for Genomic Integrity, Institute for Basic Science (IBS), Ulsan 44919, Republic of Korea; Center for Genomic Integrity, Institute for Basic Science (IBS), Ulsan 44919, Republic of Korea; Center for Genomic Integrity, Institute for Basic Science (IBS), Ulsan 44919, Republic of Korea; Center for Genomic Integrity, Institute for Basic Science (IBS), Ulsan 44919, Republic of Korea; Department of Biological Sciences, Ulsan National Institute of Science and Technology, Ulsan44919, Republic of Korea; Center for Genomic Integrity, Institute for Basic Science (IBS), Ulsan 44919, Republic of Korea; Center for Genomic Integrity, Institute for Basic Science (IBS), Ulsan 44919, Republic of Korea; Center for Genomic Integrity, Institute for Basic Science (IBS), Ulsan 44919, Republic of Korea; Center for Genomic Integrity, Institute for Basic Science (IBS), Ulsan 44919, Republic of Korea; Department of Biological Sciences, Ulsan National Institute of Science and Technology, Ulsan44919, Republic of Korea; Center for Genomic Integrity, Institute for Basic Science (IBS), Ulsan 44919, Republic of Korea; Department of Biological Sciences, Ulsan National Institute of Science and Technology, Ulsan44919, Republic of Korea; Center for Genomic Integrity, Institute for Basic Science (IBS), Ulsan 44919, Republic of Korea; Department of Biomedical Engineering, Ulsan National Institute of Science and Technology, Ulsan44919, Republic of Korea; Department of Biological Sciences, Ulsan National Institute of Science and Technology, Ulsan44919, Republic of Korea; Center for Genomic Integrity, Institute for Basic Science (IBS), Ulsan 44919, Republic of Korea; Department of Biological Sciences, Ulsan National Institute of Science and Technology, Ulsan44919, Republic of Korea; Center for Genomic Integrity, Institute for Basic Science (IBS), Ulsan 44919, Republic of Korea; Department of Biological Sciences, Ulsan National Institute of Science and Technology, Ulsan44919, Republic of Korea; Center for Genomic Integrity, Institute for Basic Science (IBS), Ulsan 44919, Republic of Korea; Department of Biomedical Engineering, Ulsan National Institute of Science and Technology, Ulsan44919, Republic of Korea

## Abstract

DNA double-strand break (DSB) repair via homologous recombination is initiated by end resection. The extent of DNA end resection determines the choice of the DSB repair pathway. Nucleases for end resection have been extensively studied. However, it is still unclear how the potential DNA structures generated by the initial short resection by MRE11-RAD50-NBS1 are recognized and recruit proteins, such as EXO1, to DSB sites to facilitate long-range resection. We found that the MSH2-MSH3 mismatch repair complex is recruited to DSB sites through interaction with the chromatin remodeling protein SMARCAD1. MSH2-MSH3 facilitates the recruitment of EXO1 for long-range resection and enhances its enzymatic activity. MSH2-MSH3 also inhibits access of POLθ, which promotes polymerase theta-mediated end-joining (TMEJ). Collectively, we present a direct role of MSH2-MSH3 in the initial stages of DSB repair by promoting end resection and influencing the DSB repair pathway by favoring homologous recombination over TMEJ.

## Graphical Abstract

**Graphical Abstract:**
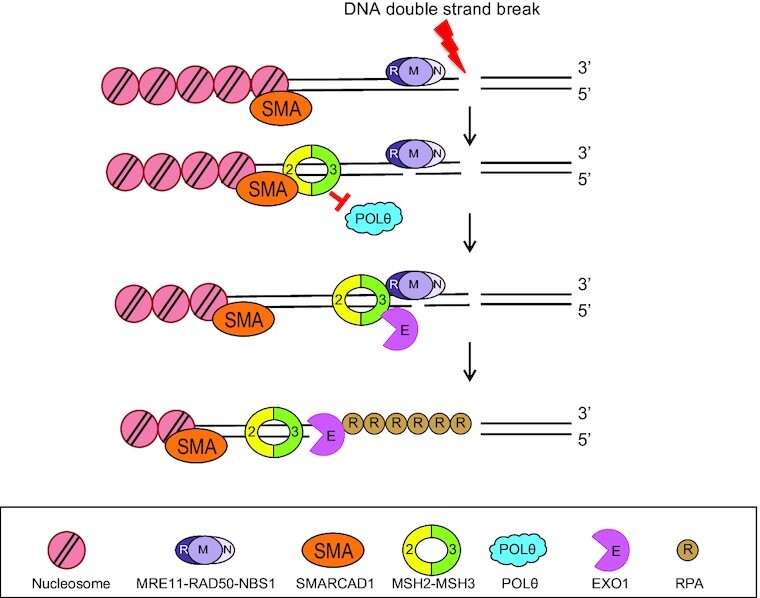
At the early step of DNA double strand break repair, SMARCAD1 recruits MSH2-MSH3 complex to inhibit POLθ-mediated end joining and promote long range end resection by EXO1 through protein-protein interaction.

## INTRODUCTION

Genome integrity is constantly challenged by DNA replication errors and diverse damaging agents, such as oxidative stress and environmental radiation (1,2). To maintain genomic stability, cells possess DNA repair mechanisms. DNA mismatch repair (MMR), which is conserved in all organisms, corrects DNA mismatches and insertion-deletion loops (IDL) resulting from DNA replication and recombination between closely related, but not identical, DNA sequences (3,4). MMR is initiated by two MMR protein complexes, depending on the nature of the DNA mismatch. In eukaryotes, the MutSα and MutSβ heterodimeric homologs composed of MSH2 (MutS Homolog 2) and MSH6 or MSH2 and MSH3, recognize mismatches of one to two nucleotides or more than two nucleotides and IDLs, respectively (5–7). After MutS homolog binds to DNA lesions, heterodimeric MutL homologs (MLH1-PostMeiotic Segregation 2 [PMS2], MLH1-PMS1 or MLH1-MLH3 in eukaryotes) are recruited with MutS homolog to enhance mismatch recognition and promote conformational changes in MutS homolog, allowing the MutL/MutS-homologs complex to slide away from mismatched DNA (8,9). Repair is then initiated by a single-stranded nick generated by MutL at a certain distance from the lesion (10,11). Exonuclease 1 (EXO1) removes the mismatch-containing strand, and correct genetic information is restored by gap filling (12,13).

DNA double-strand breaks (DSBs) are considered as one of the most threatening types of DNA damage. Since unrepaired DSBs can cause chromosome discontinuity and translocations, proper and precise DSB repair is essential for preventing genomic instability and, ultimately, tumorigenesis (1,14,15). DSBs are repaired by three major pathways: homologous recombination (HR), nonhomologous end-joining (NHEJ), and microhomology-mediated end-joining (MMEJ and also known as DNA polymerase θ [POLθ]-mediated end-joining [TMEJ]) (16). A key factor determining the pathway choice among the HR, NHEJ, and TMEJ pathways is the length of a 3′ overhang generated by DNA end resection, mediated by the nucleolytic removal of the complementary strand. The MRE11-RAD50-NBS1 (MRN) complex binds to broken DSBs, and end resection is initiated by a nick generated by MRE11 nuclease, followed by short-range resection promoted by CtIP. Further 5′-3′ resection is mediated by EXO1, together with Bloom (BLM) or Werner (WRN) helicases and the DNA2 nuclease (17–21). In case of extensive end resection across tandem DNA repeats, DSBs may also be mended by the pairing of homologous repeats, leading to the loss of interspersed sequences, a process referred to as single-strand annealing (SSA) (22). NHEJ directly ligates broken DSBs. However, small deletions may arise when broken DNA ends are degraded. TMEJ acts on partially resected DNAs and relies on the extension of short complementary DNA stretches (microhomology) by POLθ to seal broken DNA at the expense of losing genetic information (23,24). The detailed mechanisms determining whether the initially formed 3′ overhangs are further resected for HR or are used as a substrate for error-prone TMEJ remain unknown.

The MMR protein MSH2-MSH3 (MutSβ) has also been implicated in HR. MSH2-MSH3 is required for proper ATR-dependent DNA damage signaling to assist HR (25). MSH2-MSH3 acts to prevent recombination between divergent DNA sequences (26–28). MSH2-MSH3 recognizes secondary DNA structures that carry mismatches in resected DNA (29). Nevertheless, the mechanism by which the MSH2-MSH3 heterodimer is recruited to DSBs and how it contributes to the downstream steps of HR remain unclear. A possible connection may be the ‘SWI/SNF-related Matrix-Associated Actin-Dependent Regulator of Chromatin Subfamily A containing DEAD/H Box1 (SMARCAD1) protein. Resection of DSB ends is promoted by SMARCAD1 (Fun30 in yeast) in human cells (30,31). SMARCAD1 interacts with MSH2-MSH6 for proper MMR (32,33). However, it is still unclear how the interaction between SMARCAD1 and MMR proteins modulates MMR and HR. Another protein involved in MMR and HR is EXO1. How EXO1 is activated by interaction with MSH2 in MMR is known. However, it is unclear whether this interaction may play a role in HR (34–38).

Here, we initially aimed to uncover the role of MSH2-MSH3 in HR. Findings reveal that MSH2-MSH3 contributes to HR through its interaction with SMARCAD1 and EXO1. We demonstrate that SMARCAD1, MSH2-MSH3, and EXO1 are sequentially recruited to the DSB to initiate DNA end resection. MSH2-MSH3 prevents POLθ recruitment to broken DNA and inhibits the sealing of broken DNA ends through TMEJ by blocking POLθ polymerase activity on annealed microhomology sequences carrying DNA mismatch. Blockage of TMEJ facilitates error-free HR via extensive EXO1-dependent end resection.

## MATERIALS AND METHODS

### Cell culture and treatment

U2OS, HEK293T and HeLa cells were purchased from American Type Culture Collection and maintained in high glucose Dulbecco's modified Eagle's medium (DMEM; Hyclone) with 10% fetal bovine serum (FBS; Millipore) and 1% penicillin and streptomycin (Invitrogen) at 37°C and 5% CO_2_. For DNA repair assays, U2OS cells stably expressing DR-GFP (HR), SA-GFP (SSA), EJ2-GFP (TMEJ) and EJ5-GFP (NHEJ) (39–42) were grown in DMEM (Gibco) containing 10% FBS (Merck) and 2 μg/ml puromycin (Invitrogen). Human *MSH2* cDNA was PCR-amplified from human cDNA isolated from HeLa cells using TRIzol (Invitrogen) and cloned into the EGFP-C2 vector using *Sal*I and *BamH*I restriction sites, and the pcDNA3.1 myc-His A vector using *BamH*I and *Apa*I sites. SMARCAD1 cDNA (43) that was a gift from Tej K. Pandita, Baylor College of Medicine, was cloned into the EGFP-C2 vector using *Sal*I and *BamH*I restriction sites and into the pcDNA3.1 myc-His A vector using *BamH*I and *Xba*I sites. EXO1 cDNA (44) that was a gift from Zhongsheng You, Washington University School of Medicine, was cloned into the EGFP-C2 vector using *Sal*I and *BamH*I restriction sites and the pcDNA3.1 myc-His A vector using *BamH*I and *Apa*I sites. All cDNAs were confirmed by sequencing. The plasmid for expressing the active DNA polymerase fragment of POLθ (Sumo3 POLQM1) was a gift from Sylvie Doublie and Susan Wallace, University of Vermont (Addgene plasmid # 78462) (45). Full-length POLθ without a stop codon was cloned into a pcDNA-DEST47 plasmid (Invitrogen), resulting in a GFP-tagged protein at the C-terminus.

### Laser microirradiation

U2OS cells 3 × 10^5^ were plated in confocal dishes (SPL) and incubated them for one day. Then, 2 μg of each plasmid expressing GFP-MSH2, GFP-SMARCAD1, or GFP-EXO1 was transfected using Lipofectamine 3000 according to the manufacturer's instructions. Media containing plasmids with Lipofectamine were replaced with media containing 10 μM 5-bromo-2’-deoxyuridine after 4 h and incubated for 24 h. A 355 nm ultraviolet A laser was used for laser microirradiation, followed by incubation of the cells in a 37°C chamber in an atmosphere of 5% CO_2_. After each laser microirradiation, cell images were obtained every 10 s for 5 min using an LSM880 confocal microscope (Carl Zeiss). The intensity of each laser stripe was determined using Zen Blue software (Carl Zeiss). The values were normalized to baseline values. At least 10 cells were used for quantification.

### Small interfering RNA (siRNA) transfection

Each 20 nM siRNA aliquot was transfected into cells using Lipofectamine RNAiMAX reagent (Invitrogen) and incubated for 48 h. The siControl #1 (5′-CGU ACG CGG AAU ACU UCG A-3′), siControl #2 (5′-CGU ACG CGG AAU ACU UCG A-3′), siMSH2 #1 (5′-AAU CUG CAG AGU GUU GUG CUU-3′), siMSH2 #2 (5′-CGA CCA GCC AUU UUG GAG A-3′), siMSH3 #1 (5′-UCG AGU CGA AAG GAU GGA UAA-3′), siMSH3 #2 (5′-GAA AAU GAU GGG CCU GUU A-3′), siMSH6 #1 (5′-AUC GCC AUU GUU CGA GAU UUA-5′), siMSH6 #2 (5′-CUG ACA AAA UCU CCG AAG U-3′), siSMARCAD1 #1 (5′-CUC CAU GGA UUA AUU CCU U-3′), siSMARCAD1 #2 (5′-GAC GAU UGA AGA AUC CAU GCU-3′), siEXO1 #1 (5′-CAC AUG CGA CCU CUG AGA U-3′), siEXO1 #2 (5′-CAA GCC UAU UCU CGU AU-3′), and siMLH1 (5′-GUG UUC UUC UUU CUC UGU A-3′) oligonucleotides were purchased from Bioneer.

### Plasmid transfection and immunoprecipitation

HEK293T cells were seeded in 100-mm dishes and incubated for 1 day to produce approximately 60% confluent growth. Each plasmid was then transfected with Transporter 5 reagent (Polysciences), according to the manufacturer's instructions. After 24 h of incubation, cells were washed with ice-cold phosphate-buffered saline (PBS) and lysed in buffer X (100 mM Tris-HCl [pH 8.0], 250 mM NaCl, 1 mM EDTA, 1% NP-40) with protease inhibitor cocktail (cat. no. 11836170001; Merck), Benzonase (cat. no. M018S; Enzynomics), and 5 mM MgCl_2_. Cell lysates were homogenized by sonication, and insoluble debris was removed by centrifugation at 12,000 g at 4°C for 10 min. Anti-Myc primary antibody (cat. no. 9E10; Santa Cruz Biotechnology) was added to the supernatant for overnight immunoprecipitation. The immunocomplexes were pulled down with Dynabeads Protein G beads (cat. no. 10004D; Invitrogen) and washed three times with buffer X. Samples were eluted with 2× NuPAGE sample buffer (Invitrogen) and proteins were resolved by SDS-PAGE. Endogenous immunoprecipitation was performed using ∼80% confluent HEK293T cells and the following antibodies: anti-MSH2 (cat. no. ab52266; Abcam), anti-SMARCAD1 (cat. no. NB100-79835; Novus), and anti-EXO1 (cat. no. ab95012; Abcam).

### FokI assay

FokI-U2OS cells, a stable cell line with a *Fok*I restriction enzyme site, were plated in a four-well plate and transfected with control, MSH2, or MSH3 siRNA. The next day, Lipofectamine 3000 was used to transfect the cells with LacI-mCherry-FokI expression plasmid and GFP-*EXO1* or mNeon-*MRE11*. After 48 h, the transfected cells were stained with Hoechst for 15 min to visualize the nuclei. Live cell images were obtained using a model LSM880 confocal microscope (Carl Zeiss).

### Cell cycle analysis

U2OS cells were transfected in a 60-mm diameter plate with the indicated siRNAs. After 48 h, the cells were fixed with 70% (v/v) ice-cold ethanol and incubated at -20°C for 1 h. Cells were washed once with ice-cold PBS and stained with propidium iodide in fluorescence-activated cell sorting (FACS) buffer (1× PBS, 0.1% Triton X-100, 0.2 mg/ml RNase A) at 37°C for 30 min. The stained cells were analyzed using a Becton Dickinson FACSVerse flow cytometer.

### Cell-based unidentified protein interaction discovery (CUPID) assay

HEK293T cells were transfected with PKC-δ-*MSH2* and either GFP-*SMARCAD1* wild-type (WT) or GFP-*SMARCAD1*-D1 mutant plasmids. For MSH2 and EXO1 binding experiments, HEK293T cells were transfected with PKC-δ-*MSH2* and either GFP-*EXO1* WT or GFP-*EXO1* D16 mutant. After 24 h, cells were treated with 1 μM phorbol 12-myristate 13-acetate (PMA) and incubated for 5 min. PMA-treated cells were washed twice with PBS, incubated with 4% formaldehyde for 5 min, and washed with PBS. Cell images were obtained using an LSM880 confocal microscope (Carl Zeiss).

### Immunofluorescence assay

Cell samples were prepared as previously described (46). Briefly, U2OS cells plated on LabTek chamber slides (Thermo Fisher Scientific) were incubated in CSK buffer for 10 min. The cells were fixed with 4% paraformaldehyde for 20 min. Cells were incubated with the anti-Rad51 antibody (cat no. 8875; Cell Signaling Technology) or anti-RPA antibody (cat no. ab2175; Abcam) at 4°C overnight. After 30 min incubation with Alexa Fluor-conjugated secondary antibody, cells were mounted with ProLong Gold antifade reagent (Vector Laboratories). Confocal images were obtained using an LSM880 confocal microscope (Carl Zeiss). The images were analyzed using ZEN2.1 software.

### HR, SSA, NHEJ, and TMEJ assays

SceI (pCAGGS-I-SceI, also denoted pCBASce), empty vector (pCAGGS-BSKX), and dsRed vector (a gift from Jeremy Stark) were prepared as previously described (39–41). U2OS cells stably expressing DR-GFP, SA-GFP, EJ2-GFP, or EJ5-GFP plasmids were plated on a 12-well plate (1 × 10^5^ cells/well). The following day, the cells were transfected with 20 nM siRNA duplex mixed with RNAiMAX (Invitrogen) in Opti-MEM. After 24 h, a second round of transfection was performed. The following day, the cells were co-transfected with 0.5 μg of either I-SceI expression vector or empty vector, and 0.1 μg of dsRED vector (used as a transfection control) in 0.1 ml Opti-MEM containing 3 μl of Lipofectamine 3000 (Invitrogen). After 6 h, the medium was removed and replaced with the growth medium. Two days after I-SceI transfection, the percentage of GFP-positive (GFP+) cells was analyzed using a Becton Dickinson FACSVerse flow cytometer. DNA repair efficiency was calculated as described previously (42). The experiments were repeated at least three times.

### End resection assay

ER-*Asi*SI U2OS cells were prepared as previously described (47). Trypsinized cells were resuspended with 0.6% low-melting agarose (Bio-Rad) at a concentration of 1.2 × 10^7^ cells/ml. Fifty microliters of cell suspension was used to make an agar ball, which was incubated with ESP buffer (0.5 M EDTA, 2% N-lauroylsarcosine, 1 mg/ml proteinase K, 1 mM CaCl_2_, pH 8.0) at 16°C for 20 h. The agar ball was treated with HS buffer (1.85 M NaCl, 0.15 M KCl, 5 mM MgCl_2_, 2 mM EDTA, 4 mM Tris, 0.5% Triton X-100, pH 7.5) at 16°C for 20 h. Melted agar balls were incubated overnight with restriction enzyme (*BsrG*I or *Hind*III-HF; New England Biolabs). Real-time PCR was performed with restriction enzyme-treated or non-treated samples. The percentage of single-strand DNA (ssDNA) was calculated as described previously (47). Briefly, the $\Delta$Ct value was calculated by subtracting the Ct value of an untreated sample from that of a sample treated with the restriction enzyme. The ssDNA fraction was calculated (47) as $ssDNA\ fraction\ \ ( \% ) = \ (1/( {{2^{( {\Delta Ct - 1} )}} + 0.5} )) \times 100$.

### Replication protein A (RPA) retention assay

Cells were treated with 5 μM camptothecin (CPT) for 1 h, or 62.5 μM baicalein for 24 h. The trypsinized cells were transferred to a 1.5 ml tube, washed with PBS, and permeabilized with 100 μl of 0.2% Triton X-100 in PBS for 10 min on ice. After washing with 1× PBS containing 1 mg/ml bovine serum albumin (PBS-BSA), the cells were fixed and permeabilized with 100 μl BD Cytofix/Cytoperm buffer (BD Biosciences) at room temperature for 15 min. The fixed cells were washed with 0.5 ml of 1× BD Perm/Wash buffer (BD Biosciences) and suspended in 0.5 ml of 1× BD Perm/Wash buffer and sequentially incubated with anti-RPA2 and Alexa Fluor 488-secondary antibodies. Nuclei in the cells were visualized by propidium iodide staining for 15 min and analyzed using a Becton Dickinson FACSVerse flow cytometer.

### DNA preparation for *in vitro* experiments

All DNA oligomers were chemically synthesized (Bioneer) and are listed in Supplementary Table S1. Each set of oligomers was annealed by heating at 95°C for 20 min followed by slow cooling to 23°C.

### Electrophoretic mobility shift assay (EMSA)

The EMSA for MSH2-MSH3 was performed as previously described (48). All reactions were performed at 23°C. 1 nM Cy5-labeled 40 bp homoduplex, +8-loop DNA, or 58 bp flap DNA was incubated with MSH2-MSH3 at different concentrations in buffer H (20 mM HEPES [pH 7.5], 100 mM NaCl, 1 mM dithiothreitol (DTT), 2 mM MgCl_2_, 0.04 mg/ml BSA) for 5 min. For the EMSA assay for EXO1 binding to DNA substrates, 1 nM Cy5-labeled 40 bp homoduplex or + 8-loop DNA was reacted with wild-type EXO1 (WT EXO1) or EXO1 nuclease mutant D173A (Mut EXO1-D173A) at different concentrations in buffer H for 20 min, which was short enough to prevent EXO1 from digesting DNA during incubation. The reactants were then analyzed by 5% non-denaturing PAGE at 130 V for 45 min in TE buffer (45 mM Tris-HCl [pH 8.5], 0.5 mM EDTA) at 4°C. The gel was imaged by scanning Cy5 fluorescence using Typhoon RGB (Cytiva).

For SMARCAD1, 1 nM Cy5-labeled 40 bp + 8-loop DNA, 40 bp homoduplex DNA, or 58 bp flap DNA was incubated with SMARCAD1 at different concentrations in buffer H supplemented with 1 mM ATP for 15 min. To test MSH2-MSH3 recruitment by SMARCAD1, 1 nM Cy5-labeled 40 bp + 8-loop DNA, 40 bp homoduplex DNA, or 58 bp flap DNA was incubated with 4 μM or 8 μM SMARCAD1 in buffer H supplemented with 1 mM ATP for 15 min. Then MSH2-MSH3 was added at different concentrations and incubated for 5 min. To test EXO1 binding to MSH2-MSH3, 1 nM Cy5-labeled 40 bp + 8-loop DNA and 100 nM MSH2-MSH3 were incubated in buffer H for 5 min, and WT EXO1 or Mut EXO1-D173A was then added at different concentrations and further incubated for 20 min. The reactants were then analyzed by running 5% non-denaturing PAGE at 130 V for 45 min in TE buffer (45 mM Tris-HCl [pH 8.5], 0.5 mM EDTA) at 4°C. The gel was imaged by scanning Cy5 fluorescence using Typhoon RGB (Cytiva).

For MSH2-MSH6, 1 nM of Cy5-labeled 40 bp homoduplex or 40 bp GT mismatch DNA was incubated with MSH2-MSH6 at different concentrations in buffer H (20 mM HEPES [pH 7.5], 100 mM NaCl, 1 mM DTT, 2 mM MgCl_2_, and 0.04 mg/ml BSA) with 1 mM ATP for 5 min. To test MSH2-MSH6 recruitment by SMARCAD1, 1 nM of Cy5-labeled 40 bp homoduplex, 40 bp GT mismatch DNA, or 58 bp flap DNA was incubated with 4 μM or 8 μM SMARCAD1 in buffer H supplemented with 1 mM ATP for 15 min. Then MSH2-MSH6 was added at different concentrations and incubated for 5 min. To test EXO1 binding to MSH2-MSH6, 1 nM of Cy5-labeled 40 bp GT mismatch DNA and 80 nM MSH2-MSH6 were incubated in buffer H for 5 min, and WT EXOI or Mut EXO1-D173A was then added at different concentrations and further incubated for 20 min. The reactants were then analyzed by running 5% non-denaturing PAGE at 130 V for 45 min in TE buffer (45 mM Tris-HCl [pH 8.5] and 0.5 mM EDTA) at 4°C. The gel was imaged by scanning Cy5 fluorescence using Typhoon RGB (Cytiva).

All EMSA with competitors were performed with 50 nM unlabeled 40 bp homoduplex.

### EXOI nuclease activity assay

EXOI nuclease activity was tested using a previously established protocol (49). 20 nM of 40 bp homoduplex or 40 bp overhang (4-nt 3′ overhang) labeled with Cy5 (40 bp overhang) were mixed with WT EXO1 or Mut EXO1-D173A in EXOI buffer (20 mM HEPES [pH 7.5], 100 mM KCl, 1 mM DTT, 100 μg/ml BSA, 0.05% Triton X-100, 2 mM MgCl_2_) at different concentrations and incubated at 37°C for 30 min. For deproteinization, SDS and proteinase K were added to 0.2% and 0.25 μg/μl, respectively, and then further incubated at 50°C for 20 min. Digested DNA fragments analyzed by 15% non-denaturing PAGE in 0.5× TBE buffer at 200 V and 23°C for 1 h. The gel was imaged by scanning Cy5 fluorescence using Typhoon RGB (Cytiva).

To enhance EXO1 nuclease activity by MSH2-MSH3 or MSH2-MSH6, 40 nM of 90 bp flap DNA labeled with Cy5, which had a flap of dT_18_ and a 15-nt gap, was mixed with 300 nM MSH2-MSH3 or MSH2-MSH6 and incubated at 23°C for 5 min. WT EXO1 or Mut EXO1-D173A was added to the MSH2-MSH3- or MSH2-MSH6-flap DNA complex in reaction buffer (20 mM HEPES [pH 7.5], 100 mM NaCl, 1 mM DTT, 2 mM MgCl_2_, 1 mM ATP, and 40 μg/ml BSA) at different concentrations (5, 10, 20, 25, 30, and 40 nM) and incubated at 37°C for 30 min. To confirm the enhancement of the EXOI nuclease activity by MSH2-MSH3 or MSH2-MSH6, the EXO1 concentration was fixed and titrated MSH2-MSH3 or MSH2-MSH6. Cy5-labeled flap DNA (40 nM) was mixed with MSH2-MSH3 at different concentrations (10, 30, 100, 300, and 500 nM) in reaction buffer and incubated at 23°C for 5 min. 20 nM of WT EXO1 or Mut EXO1-D173A was added to the MSH2-MSH3- or MSH2-MSH6-flap DNA complexes and further incubated at 37°C for 30 min. All reactions were stopped and deproteinized by incubation with 0.2% SDS and 0.25 mg/ml proteinase K at 50°C for 20 min. Digested DNA fragments were analyzed by 15% non-denaturing PAGE in 0.5 × TBE buffer at 200 V and 23°C for 1 h. The gels were imaged using Typhoon RGB (Cytiva).

### Protein purification

Full-length human MSH2-MSH3, EXO1 WT, Mut EXO1-D173A, SMARCAD1, and MSH2-MSH6 were obtained by infecting Hi5 insect cells with amplified baculoviruses. To enhance the protein solubility of EXO1, Mut EXO1-D173A, and SMARCAD1, we added a maltose-binding protein tag to the N-terminus of the protein. After 48 h of virus infection, cells were harvested and resuspended in buffer A containing 25 mM sodium phosphate [pH 7.8], 400 mM NaCl, and 10 mM imidazole with a protease inhibitor cocktail (cat. no. 11873580001; Roche). The supernatant was applied to a HisTrap HP column (cat. no. 17524802; Cytiva), and proteins were eluted with a linear gradient of buffer B (buffer A + 400 mM imidazole). Protein peaks were collected and concentrated using an Amicon ultra-15 50 K centrifugal filter. Concentrated proteins were then applied to a HiLoad 26/600 Superdex 200 pg column (cat. no. 28989336; Cytiva) equilibrated in buffer consisting of 25 mM Tris-HCl [pH 7.5], 150 mM NaCl, and 5 mM DTT. The fractionated protein peak from each step was confirmed using SDS-PAGE. Protein concentrations were measured using the Bradford assay.

For MSH2-MSH6 purification, cells were harvested and resuspended in buffer C containing 25 mM HEPES [pH 7.5], 150 mM KCl, 0.1 mM EDTA, 10% glycerol, and 1 mM DTT with 0.1% phenylmethyl sulfonyl fluoride (PMSF) and protease inhibitor cocktail (cat. no. 11873580001; Roche). The supernatant was applied to a HiTrap Heparin HP column (cat. no. 17040601; Cytiva), and proteins were eluted with a linear salt gradient in buffer C up to 650 mM KCl. After adjusting the salt concentration of the collected sample to 150 mM KCl, we applied proteins to a HiTrap Q HP column (cat. no. 17115301; Cytiva) and eluted them with a linear salt gradient similar to that of the heparin column. Protein peaks were collected and concentrated using an Amicon ultra-15 50 K centrifugal filter. Concentrated proteins were then applied to a HiLoad 26/600 Superdex 200 pg column (cat. no. 28989336; Cytiva) equilibrated in buffer consisting of 25 mM HEPES [pH 7.5], 100 mM KCl, 0.1 mM EDTA, 10% glycerol, and 1 mM DTT. The fractionated protein peak from each step was confirmed by SDS-PAGE. Protein concentrations were measured using the Bradford assay.

### *In vitro* immunoprecipitation

The primary antibody was added to Dynabead protein G (cat. no. 10004D; Invitrogen) for 2 h. The purified proteins were incubated with primary antibody-conjugated Dynabead for 1 h. After washing three times with buffer X (100 mM Tris-HCl [pH 8.0], 250 mM NaCl, 1 mM EDTA, 1% NP-40, and 0.1% Triton X-100), we added the second protein to the first protein-bound Dynabead for 1 h. Immunocomplexes were pulled down with Dynabead protein G, washed three times with buffer X, and then subjected to SDS-PAGE and western blotting. Proteins were visualized using enhanced chemiluminescence (ECL) buffer (WBKL S0100; Millipore). Signals were detected using an Amersham Imager 680 chemiluminescence image analyzer (Cytiva,). The target proteins were immunoprecipitated with each antibody (anti-MSH2, cat. no. ab52266, Abcam; anti-SMARCAD1, NB100-79835, Novus; anti-EXO1, ab95012, Abcam).

### DNA polymerase assays

Active POLθ DNA polymerase fragment was expressed from the Sumo3 POLQM1 plasmid and purified as previously described (45). The Klenow Fragment (3′→5′ exo-) was purchased from NEB. POLθ was diluted in buffer containing 37.5 mM Tris-HCl [pH 8.0], 40 mM NaCl, 2.5 mM DTT, 6.25% glycerol, 0.0125% Triton X-100, and 0.125% BSA. The Klenow Fragment (3′→5′ exo-) was diluted in buffer containing 50 mM Tris-HCl [pH 7.5], 1 mM DTT, 50% glycerol, and 0.1 mM EDTA. PAGE-purified oligonucleotides were purchased from IDT. The primer (5′-AAAAAAAAATATGATG) was 5′-labeled using polynucleotide kinase and [γ-^32^P]dATP. The 5′-^32^P-labeled primer was annealed to template oligonucleotides containing no or 2 bp mismatch bases in different positions as follows (2 bp mismatch underlined): no-mismatch template: TTTTTTTTTATACTACTACTACGACTGCTC-5; MM-1,2 template: TTTTTTTTTATACTGTTACTACGACTGCTC-5′; MM-3,4 template: TTTTTTTTTATATCACTACTACGACTGCTC-5′; and MM-5,6 template: TTTTTTTTTACGCTACTACTACGACTGCTC-5′ POLθ reaction mixtures (10 μl) containing 30 mM Tris-HCl [pH 8.0], 0.5% glycerol, 0.4 mM DTT, 0.02% BSA, 20 mM MgCl_2_, 100 μM of each dNTP, and 100 nM of the primer-template or primer. Klenow Fragment (3′→5′ exo-) reaction mixtures (10 μl) contained 50 mM Tris-HCl [pH 7.2], 0.1 mM DTT, 10 mM MgSO_4_, 100 μM of each dNTP, and 100 nM of the primer-template. After incubation at 37°C for 10 min, the reactions were terminated by adding 10 μl formamide stop buffer (98% formamide, 10 mM EDTA [pH 8.0], 0.025% xylene cyanol FF, and 0.025% bromophenol blue) and heating at 95°C for 3 min. The products were electrophoresed on denaturing 20% polyacrylamide (7 M urea gel) and analyzed using Typhoon RGB (Amersham). For the MSH2-MSH3 derivatives assay, we first incubated MSH2-MSH3 derivatives with DNA substrates in the reaction buffer without POLθ at room temperature (25°C) for 20 min and then incubated with POLθ at 37°C for 10 min.

### Determination of termination probability and amount of full-length extension

The termination probability at position N was defined as the band intensity at N divided by the total intensity of all bands ≥ *N*, as previously described (50). The quantification of full-length extension products was defined as the fully extended band intensity divided by the intensity of all bands ≥ *N*0 (primer position).

### Targeted deep sequencing

We transfected 20 nM of control or MSH2 siRNA into 1.5 × 10^6^ HEK293T cells in a 10 cm dish. After 24 h of incubation, we transfected 4 μg of p3s-Cas9-HN and 6 μg of each plasmid expressing mCherry-gRNA (targeting CEL and non-targeting gRNA control 1 and control 2) using Lipofectamine 3000, according to the manufacturer's instructions. Cells were incubated for 2 days and only Cas9- and mCherry-gRNA-transfected cells were sorted by FACS using a FACSAria Fusion device (BD Bioscience). Cells (2 × 10^5^) expressing the mCherry signal were sorted, and genomic DNA was extracted using the QiAamp DNA Mini Kit (QIAGEN) according to the manufacturer's instructions. To measure the mutation frequency at the CRISPR-Cas9 induced DSB site by genomic sequencing, we performed nested PCR with 200 ng of genomic DNA using each primer: CEL F1: 5′-TGTGGACATCTTCAAGGGCA-3′, CEL R1: 5′-AGATCATAACGGGCAGGTCC-3′; CEL F2: 5′-GTGACTGGAGTTCAGACGTGTGCTCTTCCGATCT CCTTCCTCATGCCAACTCCT-3′, CEL R2: 5′-ACACTCTTTCCCTACACGACGCTCTTCCGATCTCTCAAGCCAGGAGTAGACCC-3′. Pooled PCR products were sequenced using a NextSeq 500/550 Mid Output kit v2.5, with 300 cycles (Illumina). Sequencing reads were analyzed using CRISPRpic (51) software for the frequency of microhomology-mediated repair.

## RESULTS

### Depletion of MSH2 and MSH3 decreases HR

**Figure 1.**
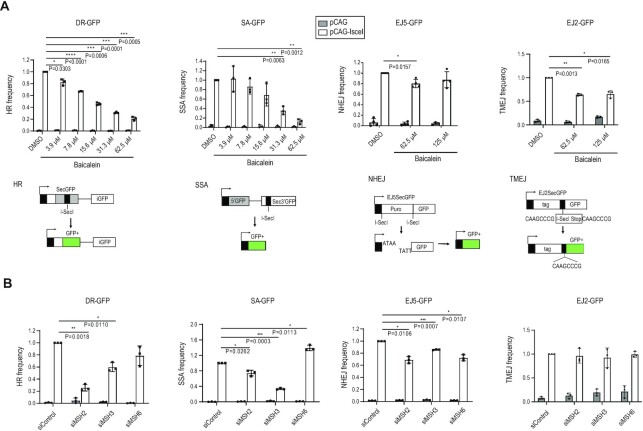
MSH2 and MSH3 functions in homologous recombination (HR) and single-strand annealing (SSA). (**A**) U2OS cells stably expressing DR-GFP, SA-GFP, EJ5-GFP, and EJ2-GFP constructs were treated with dimethyl sulfoxide or the indicated doses of baicalein for 24 h. The efficiency of HR, SSA, NHEJ, and TMEJ, respectively, was determined by scoring the percentage of GFP-positive cells. Schematic diagram of each construct is shown below. (**B**) The efficiency of HR, SSA, NHEJ, and TMEJ was measured after transfection of control, MSH2, MSH3, or MSH6 siRNA. Data are presented as mean ± standard deviation (*n* = 3, independent cell culture). *P*-values were calculated by two-tailed Student's *t*-test.

We previously found that the natural compound baicalein inhibits MMR (52) and selectively kills MMR-deficient cancer cells. Baicalein is derived from *Scutellaria baicalensis* and is widely used in traditional Chinese medicine (53). Given the reported interconnection between the MMR and DSB repair pathways, we hypothesized that baicalein influences DSB repair. To explore this, U2OS cells were treated with increasing concentrations of baicalein, and the frequencies of HR, SSA, NHEJ, and TMEJ were measured. Using established reporter assays based on the restoration of GFP expression (39–41), we observed that the frequencies of HR and SSA were decreased by baicalein treatment in a dose-dependent manner. In contrast, NHEJ and TMEJ were not significantly affected (Figure 1A). Since baicalein binds to MutS complexes (both MSH2-MSH3 and MSH2-MSH6), we hypothesized that MSH2-MSH3 and MSH2-MSH6 may mediate the effect of baicalein on HR and SSA. To test this hypothesis, we depleted the components of the MSH2-MSH6 (MutSα) and MSH2-MSH3 (MutSβ) complexes (Supplementary Figure S1A) and assessed the frequencies of HR, SSA, NHEJ and TMEJ. Knockdown of MSH2 or MSH3 strongly reduced the frequencies of HR and SSA, consistent with a previous report that MSH2-MSH3 plays a role in removing nonhomologous tails during SSA in yeast (54), whereas knockdown of the MutSα-specific subunit, MSH6, did not (Figure 1B and Supplementary Figure S1B). Consistent with the baicalein treatment results, depletion of MSH2, MSH3 or MSH6 proteins had only a small effect on NHEJ and TMEJ (Figure 1B). To determine whether baicalein treatment resulted in additional HR defects in MSH2, MSH3 or MSH6 knockdown cells, baicalein was co-treated with each knockdown cell. After treatment with baicalein, additional HR defects were observed in each knockdown cell (Supplementary Figure S1C). We tested whether MSH6 knockdown affects the level of the endogenous MSH2-MSH3 complex by immunoprecipitation with an MSH3 antibody in MSH6 depleted cells. Total amount of the MSH2-MSH3 complex was not changed by MSH6 knockdown (Supplementary Figure S1D). These data suggest that MSH6 knockdown does not change the level of the MSH2-MSH3 complex, but does affect the MSH2-MSH6 complex. In summary, consistent with a previous report (25), the reduction in HR and SSA frequencies conferred by MSH2 or MSH3 depletion correlated with the effect of baicalein treatment observed in this study. Thus, MSH2-MSH3 (MutSβ), not MSH2-MSH6 (MutSα), contributes to HR and SSA.

### Depletion of MSH2 and MSH3 suppresses DNA end resection

**Figure 2.**
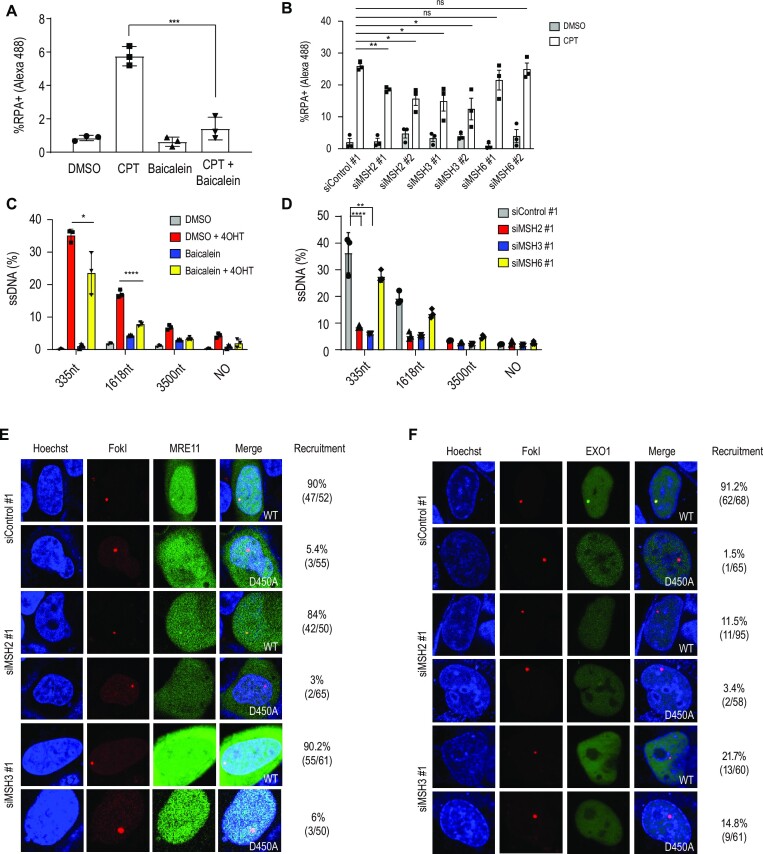
MSH2 and MSH3 are required for DNA end resection. (**A**) RPA chromatin association was measured after treatment with camptothecin (CPT), baicalein, and the combination of CPT with baicalein. Treatment with dimethyl sulfoxide (DMSO) served as a control. Cells were treated with 62.5 μM baicalein for 24 h. CPT (5 μM) was incubated for 1 h. Harvested cells were fixed and incubated with RPA2 antibody and the percentage of RPA2 positive cells was analyzed by FACS analysis. (**B**) RNAi depletion was performed for 48 h, and the proportion of RPA2 positive cells was determined by FACS analysis. (**C**) DSBs were induced in ER-*Asi*SI cells by a 4-h incubation of 4-OHT after treating cells for 24 h with 62.5 μM baicalein. Resected DNA was quantified by qPCR after restriction digests (or mock digests) with enzymes cutting double-stranded (not single-stranded) DNA, 335, 1618 and 3500 nucleotides from the *Asi*SI-induced DSBs. The percentage of amplified ssDNA in relation to DNA amplified from mock treated double-stranded DNA is shown. (**D**) ER-*Asi*SI cells were transfected with indicated siRNAs and incubated 48 h. After 4 h of 4-OHT treatment, cells were harvested and subjected to the end resection analyses. Data present mean ± standard deviation (*n* = 3, independent cell culture). *P*-values were calculated by two-tailed Student's *t*-test. (**E**) mNeon-MRE11 recruitment to *Fok*I induced DSB sites was measured in control, MSH2, or MSH3 knocked down cells. (**F**) GFP-EXO1 recruitment to *Fok*I induced DSB sites was measured in control cells and upon MSH2 or MSH3 depletion. U2OS cells co-transfected with GFP-EXO1 and *Fok*I WT or D450A mutant were incubated with Hoechst for 10 min to visualize the nuclei. Cells were then incubated in CO_2_ independent media, and live cell confocal microscopy images were acquired. EXO1 recruitment to DSB sites was quantified (right column) by calculating the proportion of cells showing colocalization of GFP-EXO1 fusion at the DSB site demarcated by the mCherry fusion.

Given that HR and SSA were decreased upon MSH2 or MSH3 depletion, we determined which step in the HR pathway depended on MSH2 or MSH3. We measured the extent of DNA end resection by assessing RPA2 loading onto resected ssDNA upon treatment with camptothecin using FACS analysis (55) and foci formation. The chromatin-bound portion of RPA was reduced upon baicalein treatment (Figure 2A) or depletion of MSH2 or MSH3 (Figure 2B and Supplementary Figure S1E). To directly measure the efficiency of DNA end resection, we used ER-*Asi*SI U2OS cells that generate DSBs by the induction of the *Asi*SI restriction nuclease upon 4-OHT treatment (47). The extent of resection was measured by qPCR to assess the amplification from the resected ssDNA compared to the corresponding dsDNA. The resection assay can measure resected ssDNA because un-resected double-stranded DNAs digested with restriction enzyme can no longer be used as a template for PCR amplification. Thus, only resected ssDNA resistant to restriction enzymes can be detected by real-time PCR amplification. We obtained the cycle threshold (Ct) value for each sample using real-time PCR. The calculation, a $\Delta$Ct value was calculated by subtracting the Ct value of an untreated sample from the Ct value of a sample treated with the restriction enzyme. We then calculated the ssDNA fraction (%) using the following equation: $ssDNA\ fraction\ \ ( \% ) = \ (1/( {{2^{( {\Delta Ct - 1} )}} + 0.5} )) \times 100$ (Supplementary Figure S1F) (47). Induction of *Asi*SI expression resulted in extensive end resection, as measured by the amplification of 335 and 1618 bp fragments, which was blocked by baicalein treatment (Figure 2C). Consistent with the baicalein results, end resection was significantly reduced in MSH2 or MSH3 depleted cells. Consistent with our HR analysis, no effect was observed upon MSH6 (MutSα) depletion (Figure 2D).

### MSH2-MSH3 interacts with EXO1 to promote end resection activity

We next monitored the recruitment of MRE11 and EXO1 to DSB to determine the step of DNA end resection facilitated by MSH2-MSH3. DSBs are induced and visualized using a fusion protein comprising *Fok*I, the lac repressor, and mCherry, where breaks can be targeted to lac operator repeats in U2OS cells (56). MRE11 recruitment to DSBs was not affected in control, MSH2, or MSH3 siRNA-treated cells (Figure 2E), suggesting that MSH2-MSH3 is not essential for MRE11 recruitment to DSBs. In contrast, EXO1 recruitment to DSB sites was significantly decreased in MSH2 or MSH3 knockdown cells (Figure 2F). MRE11 and EXO1 recruitment to DSB was not observed when a catalytically inactive *Fok*I D450A nuclease was used (Figures 2E, F). Collectively, the MSH2-MSH3 complex is required for recruiting EXO1, but not MRE11, to DSBs.

**Figure 3.**
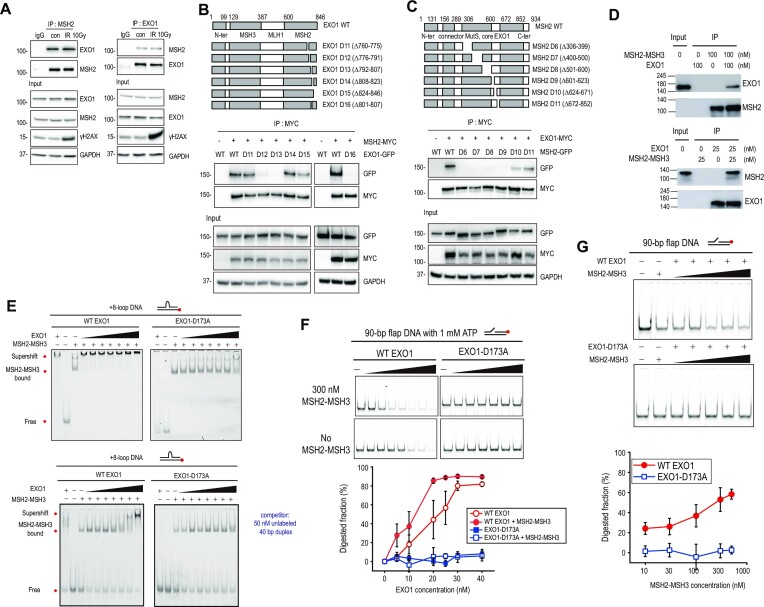
MSH2 interaction with EXO1 enhances EXO1 activity. (**A**) For endogenous immunoprecipitation, HEK293T cells were not irradiated or irradiated with 10 Gy of ionizing radiation. Cell extracts were incubated with anti-IgG, anti-MSH2, or anti-EXO1 antibody. Proteins immunoprecipitated with Dynabeads Protein G were analyzed by western blot. For 10 Gy exposure, γH2AX was used as a DNA damage marker. (**B**) Diagram of EXO1 WT and EXO1 deletion mutants. HEK293T cells were co-transfected with myc-MSH2 WT and GFP-EXO1 or GFP-EXO1 deletion mutants. Interaction of each EXO1 deletion mutant with MSH2 was determined by immunoprecipitation with MYC antibody. (**C**) Diagram of MSH2 WT and MSH2 deletion mutants. HEK293T cells were co-transfected with myc-EXO1 WT and each GFP-MSH2 deletion mutant. (**D**) Immunoprecipitation assays for purified EXO1 and MSH2-MSH3 proteins. Indicated antibodies were used for western blotting. (**E**) EMSA for MSH2-MSH3 and EXO1. MSH2-MSH3 (100 nM) was bound to +8-loop DNA, and WT EXO1 (left) or EXO1 nuclease mutant (Mut EXO1-D173A) (right), and in the absence (top) or presence (bottom) of competitor was titrated (0, 10, 15, 20, 25, 30, 40, and 80 nM). (**F**) Nuclease activity of EXO1 in the presence or absence of MSH2-MSH3 with 1 mM ATP. DNA (40 nM) with 90 bp flap DNA was reacted with WT EXO1 or Mut EXO1-D173A at different concentrations (0, 5, 10, 20, 25, 30, and 40 nM) in the presence (top) or absence (bottom) of 300 nM MSH2-MSH3. Quantification is shown below the gel images. Error bars represent standard error determined from triplicate samples. (**G**) Nuclease activity of EXO1 in the titration of MSH2-MSH3. 40 nM DNA with 90 bp flap DNA was reacted with 20 nM WT EXO1 (top) or Mut EXO1-D173A (middle) at different concentrations (0, 10, 30, 100, 300, and 500 nM) of MSH2-MSH3. The EXO1 nuclease activity was quantified (bottom). Error bars were obtained from standard error in triplicate.

EXO1 interacts with MSH2 through the C-terminal region of EXO1 (38). We first confirmed this interaction by immunoprecipitation of endogenous proteins. As previously observed, MSH2 and EXO1 interacted with each other (Figure 3A). This interaction was not changed by ionizing radiation treatment (Figure 3A). Using a series of GFP-tagged EXO1 deletion mutants (EXO1 D1 to D4) spanning the entire protein and myc-tagged MSH2, we confirmed that the C-terminal amino acid (aa) residues 600–846 of EXO1 are required for MSH2 binding (Supplementary Figure S2A) (38). To further investigate how MSH2 regulates EXO1 recruitment to DSB sites, we used a series of small C-terminal deletions and narrowed down the minimal MSH2 binding domain of EXO1 to aa residues from 801 to 807 (Figure 3B). Conversely, the minimal MSH2 domain required for EXO1 binding was determined to be aa residues from 306 to 623 (Supplementary Figure S2B, Figure 3C). This domain contains the MutS core domain and is a part of the previously annotated EXO1 binding domain (38). The requirement of the EXO1 C-terminal residues from 801 to 807 for MSH2 binding *in vivo* was further confirmed by CUPID assays (57). For this assay, a PKC-δ domain was fused to mRFP-MSH2 and employed to tether the fusion protein to the nuclear membrane upon phorbol 12-myristate 13-acetate treatment, which resulted in the localization of EXO1, but not EXO1 D16 (Δ801–807) to the nuclear membrane (Supplementary Figure S2C).

To investigate the direct interactions between MSH2-MSH3 and EXO1 *in vitro*, all proteins were purified (Supplementary Figure S2D), and the activities of the purified proteins were tested (Supplementary Figures S2E, F). We then confirmed that purified MSH2-MSH3 and EXO1 directly bind each other *in vitro* (Figure 3D). Consistent with a previous work, MSH2-MSH3 showed a higher binding affinity to an oligonucleotide substrate carrying an 8-nt loop (+8-loop DNA) compared to a corresponding homoduplex oligonucleotide substrate (Supplementary Figure S2E) (48). Wild-type EXO1 (WT EXO1) displayed the expected nuclease activity when served with a 40 bp DNA double-stranded substrate carrying a 4-nt single-stranded 3′ overhang (Supplementary Figure S2F). However, WT EXO1 did not show exonuclease activity for blunt end DNA compared to the 3′ overhang DNA (Supplementary Figure S2F). In addition, the EXO1 nuclease mutant (Mut EXO1-D173A) did not digest any type of DNA (Supplementary Figure S2F).

Having confirmed the functionality of *in vitro* purified MSH2-MSH3 and EXO1 proteins, we investigated whether MSH2-MSH3 can facilitate EXO1 recruitment to DNA substrates. Supershift assays were performed by adding EXO1 to a +8-loop-containing oligonucleotide substrate bound to MSH2-MSH3, and a dose-dependent supershift was observed (Figure 3E). Supershift by WT EXO1 was observed at a lower concentration (∼15 nM) in the presence of MSH2-MSH3, while WT EXO1 bound to the same DNA substrate only at a higher concentration (∼80 nM). For Mut EXO1-D173A, the supershift occurred at a higher concentration (∼40 nM) than that for WT EXO1, whereas Mut EXO1-D173A alone did not bind to the DNA. Taken together, MSH2-MSH3 promoted the association of WT EXO1 and Mut EXO1-D173A with DNA (Figure 3E and Supplementary Figure S2G).

We then investigated how the interaction between MSH2-MSH3 and EXO1 contributes to DNA end resection. It has been reported that MSH2-MSH6 enhances EXO1 activity in MMR (58). Thus, we tested whether MSH2-MSH3 also enhances EXO1 nuclease activity. In the presence of ATP, MSH2-MSH3 enhanced DNA degradation by WT EXO1 (Figure 3F). In addition, as the MSH2-MSH3 concentration increased at a fixed WT EXO1 concentration, more DNA was digested by WT EXO1 (Figure 3G). In contrast, in the absence of ATP, DNA degradation by WT EXO1 was slightly increased by MSH2-MSH3, indicating that ATP is important for the enhancement of WT EXO1 nuclease activity (Supplementary Figure S2H). MSH2-MSH3 did not enhance DNA digestion of catalytically inactive Mut EXO1-D173A, regardless of ATP (Figures 3F-G, and Supplementary Figure S2H). Collectively, our data suggest that EXO1 recruitment by MSH2-MSH3 enhances end resection (Figure 3F, G).

### SMARCAD1 directly interacts with MSH2-MSH3

**Figure 4.**
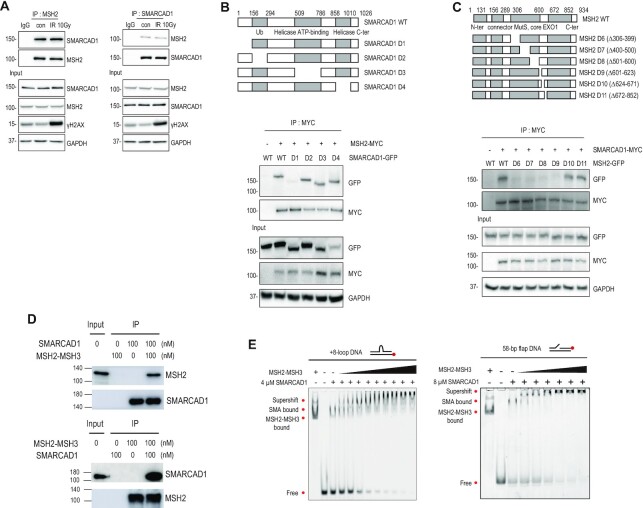
SMARCAD1 directly interacts with MSH2. (**A**) HEK293T cells were irradiated with 0 or 10 Gy. Extracts were immunoprecipitated with IgG, MSH2, or SMARCAD1 antibody. (**B**) Diagram of SMARCAD1 WT and SMARCAD1 deletion mutants. Cells were co-transfected with myc-MSH2 WT and GFP-SMARCAD1 WT or GFP-SMARCAD1 deletion mutants. Interaction of SMARCAD1 deletion mutants with MSH2 was determined by immunoprecipitation with MYC antibody. (**C**) Diagram of MSH2 WT and MSH2 deletion mutants. Myc-SMARCAD1 WT was co-transfected with GFP-MSH2 WT or GFP-MSH2 deletion mutants. (**D**) Immunoprecipitation assays for purified SMARCAD1 and MSH2-MSH3 proteins. Indicated antibodies were used for western blotting. (**E**) EMSA for SMARCAD1 and MSH2-MSH3. SMARCAD1 (4 μM) was bound to +8-loop DNA and titrated with MSH2-MSH3 (0, 10, 20, 40, 80, 100, 150, 200, 300, and 400 nM, left). 8 μM SMARCAD1 was bound to 58 bp flap DNA, and then titrated with MSH2-MSH3 (0, 10, 20, 40, 80, 100, and 150 nM, right).

MSH2-MSH3 preferentially recognizes small loop structures. Since resected DSBs do not have small loops, we investigated how MSH2-MSH3 might be recruited to DSB sites to facilitate EXO1 recruitment for end resection. We hypothesized that the protein(s) interacting with MSH2-MSH3 would help recruit MSH2-MSH3 to DSBs. The chromatin remodeler SMARCAD1 interacts with MSH2-MSH6 (32,33) and is reported to be localized at DSBs and facilitate end resection in yeast and human cells (30,43). We first re-examined whether SMARCAD1 interacts with MSH2 in HEK293T cells. Reciprocal pull-down of endogenous MSH2 and SMARCAD1 was observed using immunoprecipitation (Figure 4A), which was not changed by ionizing radiation treatment. To identify the MSH2 binding domain in SMARCAD1, we generated GFP-tagged SMARCAD1 WT and a series of deletion mutants (D1 to D4) spanning the entire protein and assessed its association with myc-MSH2 by immunoprecipitation. Bioinformatic analysis predicted that SMARCAD1 has a potential MSH2 binding domain, termed SHIP box, in its N-terminus (aa residues 5–11) (37). Our domain analysis experimentally confirmed that the N-terminus of SMARCAD1 is required for MSH2 binding, with the SMARCAD1 D1 (Δ1–156) deletion being unable to bind MSH2 (Figure 4B). Conversely, in co-transfection experiments with a series of MSH2 deletion mutations, we narrowed down the minimal SMARCAD1 binding domain in MSH2 to aa residues from 306 to 623 (Supplementary Figure S3A and Figure 4C), which were the same region of MSH2 interacting with EXO1 (Figure 3C), suggesting that SMARCAD1 and EXO1 bind to the same region of MSH2. MSH2 D6 to D9 deletion mutants spanning aa residues from 306 to 623 lost their ability to interact with EXO1 and SMARCAD1. These MSH2 deletion mutants were still able to assemble an MSH2-MSH3 heterodimer complex with MSH3 (Supplementary Figure S3B). No interaction between SMARCAD1 and EXO1 was evident by endogenous immunoprecipitation (Supplementary Figure S3C). The interaction between MSH2 and SMARCAD1 *in vivo* was confirmed by CUPID analysis (Supplementary Figure S3D).

To determine whether SMARCAD1 directly interacts with MSH2-MSH3, *in vitro* IP assays using purified proteins were performed, where purified SMARCAD1 and MSH2-MSH3 directly bind to each other *in vitro* (Figure 4D). Then we conducted EMSA to examine if SMARCAD1 facilitates MSH2-MSH3 recruitment. To mimic several possible DNA structures that could be generated after DSB, we designed and used a flap or loop DNA sequence for EMSA (Figure 4E and Supplementary Figure S3E). After SMARCAD1 was incubated with +8-loop DNA, MSH2-MSH3 was added. As the MSH2-MSH3 concentration increased, the SMARCAD1-DNA band progressively disappeared and a supershifted band emerged (Figure 4E and Supplementary Figure S3E). The band supershifted by MSH2-MSH3 started to appear at a concentration of 10 nM, whereas binding of MSH2-MSH3 alone to +8-loop DNA occurred at 80 nM (Supplementary Figure S2E). When SMARCAD1 was pre-incubated with flap DNA, MSH2-MSH3 bound to the flap DNA at a lower concentration (10 nM MSH2-MSH3) compared to the absence of SMARCAD1 (80 nM MSH2-MSH3) (Figure 4E and Supplementary Figure S3E). Similarly, SMARCAD1 enhanced the recruitment of MSH2-MSH3 to homoduplex DNA (Supplementary Figure S3E). Thus, the binding affinity of MSH2-MSH3 to DNA substrates was enhanced by approximately eight times in the presence of SMARCAD1. The finding supports the view that SMARCAD1 recruits MSH2-MSH3 and forms a complex on DNA regardless of the type of DNA substrate.

### Interdependence of SMARCAD1, MSH2, and EXO1 localization at DNA damage sites

**Figure 5.**
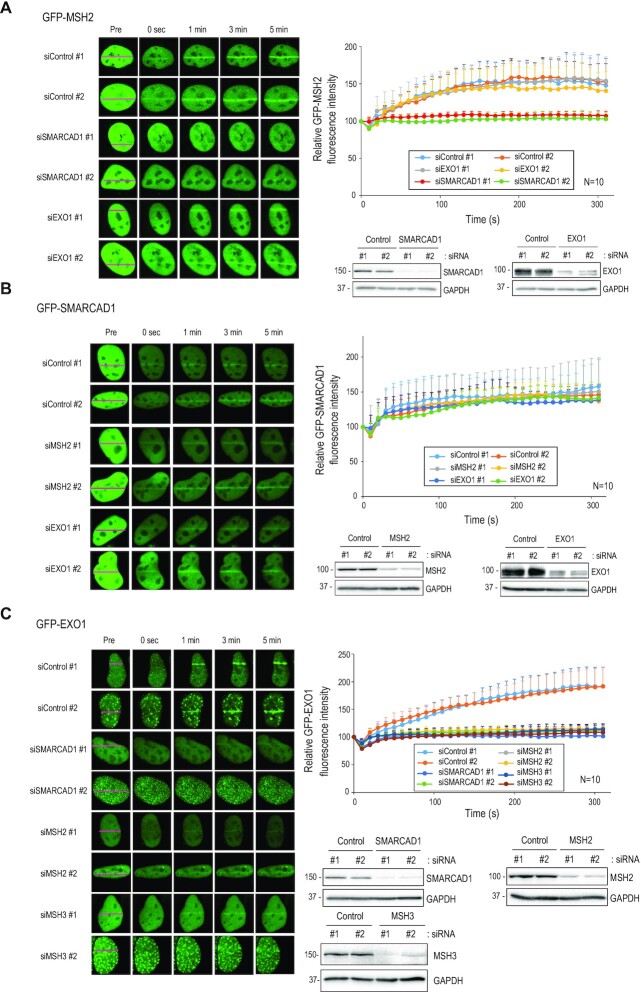
SMARCAD1, MSH2 and EXO1 move to DSB sites in an orderly manner. (**A**–**C**) U2OS cells were transfected with control or indicated siRNA. After 24 h, cells were transfected with GFP-MSH2 (**A**), GFP-SMARCAD1 (**B**), and GFP-EXO1 (**C**). Transfected cells were incubated with 10 μM of BrdU for 24 h. Cell images were taken every 10 s after microirradiation for 5 min by confocal microscopy. Data are presented as mean + standard deviation (*n* = 10).

To determine the interdependency of SMARCAD1, MSH2, and EXO1 recruitment to DNA damage sites, GFP-tagged versions of these genes were transfected into U2OS cells and their recruitment to stripes irradiated with a 355 nm laser was determined. GFP-MSH2 accumulated at microirradiation sites within 1 min of irradiation. The accumulation was compromised by deletion of SMARCAD1, but not EXO1 (Figure 5A). Depletion of MSH2 or EXO1 did not alter the recruitment of SMARCAD1 to sites of damage (Figure 5B). Finally, EXO1 recruitment was dramatically reduced in the absence of MSH2, MSH3, or SMARCAD1, but not in MSH6 knockdown cells (Figure 5C and Supplementary Figure S4A). Results of MSH2 and EXO1 recruitment in HeLa cells were similar (Supplementary Figure S4B). Taken together, these data indicate that SMARCAD1 is required for MSH2 recruitment, which in turn is needed for EXO1recruitment to DSB sites.

Since MRE11 recruitment to DSB was not dependent on MSH2 (Figure 2E), we examined the interaction between MRE11 and SMARCAD1. MRE11 recruitment to the microirradiation-induced DSB sites occurred normally in control, MSH2, or SMARCAD1 knockdown cells (Supplementary Figure S4C), indicating that MRE11 recruitment to DSB occurs independently of SMARCAD1 and MSH2. MMR proteins have been suggested to function in rejecting heteroduplex DNA with imperfect matches during later stages of HR (26–28). Formation of heteroduplex DNA requires RAD51-dependent strand invasion (59,60). Thus, we investigated whether MSH2-MSH3 recruitment to DSB depends on RAD51. Cells were treated with the RAD51 inhibitor B02 for 4 h and MSH2 recruitment to microirradiation-induced DSB was monitored. MSH2 accumulation to DSBs was not influenced by the RAD51 inhibitor and RAD51 depletion (Supplementary Figure S4D), indicating that MSH2 acts upstream of RAD51 and locates to DSBs before the strand invasion stage of the HR. Knockdown of MLH1 did not affect HR, end resection, and EXO1 recruitment to DSB, suggesting that MSH2-MSH3 in MMR is a major protein involved in the recruitment of EXO1 for the end resection of HR repair (Supplementary Figure S4E). Recently, MLH1 deficiency leads to the hyperactivation of EXO1 resulting in excessive long-range resection (61). In the previous study, ER-*Asi*SI and RPA foci assays were used for end resection with IR, while we used RPA foci assay with IR or CPT (Supplementary FigureS4E). For RPA foci assay, we measured RPA foci that were generated in 2 h after IR or 1 h after CPT treatment, whereas the previous study measured RPA foci that in 24 h after IR treatment. Our result provides the initial step of end resection for recruitment of EXO1 by MSH2-MSH3, which is different from the role of MLH1 in the termination of end resection.

### SMARCAD1-MSH2-EXO1 recruitment is important for HR

**Figure 6.**
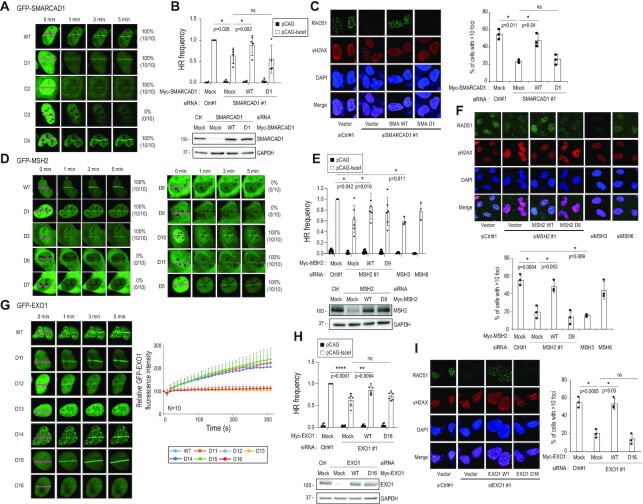
SMARCAD1, MSH2, and EXO1 are required for HR. (**A**, **D**, **G**) U2OS cells were transfected with indicated GFP-tagged construct. The series of GFP-SMARCAD1 (**A**), GFP-MSH2 (**D**) or GFP-EXO1 (**G**) transfected U2OS cells were microirradiated and their recruitment to microirradiation-induced DSBs was monitored by confocal microscopy (*n* = 10). (**G**) Data are presented as mean + standard deviation (*n* = 10). (**B**, **E**, **H**) HR frequency was measured in DR-GFP expressing U2OS cells. Cells were transfected with indicated siRNAs and siRNA-resistant DNA constructs. Data are presented as mean ± standard deviation. (**B**) *n* = 4, (**E**) *n* = 6, and (**H**) *n* = 6 independent cell culture. We adopted the MSH3 and MSH6 data from Figure 1B. (**C**, **F**, **I**) U2OS cells were transfected with indicated siRNAs and DNA constructs. Transfected cells were irradiated with 10 Gy and RAD51 foci in the nucleus were counted by confocal microscopy (*n* = 3, independent cell culture). Data are presented as mean ± standard deviation. *P*-values were calculated by two-tailed Student's *t*-test.

Next, we assessed the requirement of various SMARCAD1 domains for recruitment to the microirradiated sites. The SMARCAD1 D1 deletion mutant, which does not interact with MSH2, was localized to sites of DNA damage, similar to WT (Figure 6A). Interestingly, recruitment of the SMARCAD1 D3 deletion mutant, which lacks the DNA helicase and ATP binding domain, was decreased. The finding suggests that DNA helicase activity is important for DNA binding (Figure 6A). To directly test whether SMARCAD1 is required for HR, reporter-based HR assays were performed (see Figure 1). SMARCAD1 depletion decreased HR frequency (Figure 6B), which was complemented by transfection with an RNA interference (RNAi)-resistant full-length SMARCAD1, but not with the siRNA-resistant SMARCAD1 D1 mutant (Figure 6B). Consistent with the results of the HR assay, RAD51 foci were formed after irradiation with 10 Gy in the presence of WT SMARCAD1. However, foci formation was attenuated in the absence of SMARCAD1 or in the presence of the MSH2-interaction mutant D1 of SMARCAD1 (Figure 6C). These data show that SMARCAD1-dependent MSH2 recruitment is required for a proficient HR.

We next examined the recruitment of WT and a series of deletion mutants of GFP-MSH2, including mutants D6 to D9, spanning aa residues from 306 to 623 of MSH2 required for SMARCAD1 and EXO1 binding. WT MSH2 was recruited to microirradiated stripes, but EXO1- and SMARCAD1-binding defective MSH2 D6 to D9 mutants were not recruited (Figure 6D). MSH2 D6 to D9 deletion proteins could move into the nucleus with a nuclear localization signal, excluding the possibility that D6 to D9 MSH2 failed to move to the DSB due to its incapability to enter the nucleus (Supplementary Figure S5A). Thus, MSH2 recruitment to DSBs depends on SMARCAD1. By measuring the effect of MSH2 depletion on HR activity and RAD51 foci formation upon irradiation, it was confirmed that HR was reduced by MSH2 depletion (Figure 6E). MSH2 depletion could be rescued by expressing siRNA-resistant full-length MSH2, but not by the SMARCAD1 binding defective MSH2 D9 mutant (Figures 6E, F). Collectively, MSH2 recruitment to DNA damage depends on SMARCAD1 and is important for a proficient HR.

Lastly, to determine whether EXO1 recruitment to DSBs requires MSH2, we examined GFP-EXO1 recruitment to microirradiated stripes, HR activity, and RAD51 foci formation with GFP-tagged WT EXO1, MSH2-binding proficient EXO1 mutants (D11, D14, and D15), and MSH2-binding deficient EXO1 mutants (D12, D13, and D16) (Figure 3B and Figures 6G-I). WT, D11, D14, and D15 GFP-EXO1 were recruited to microirradiated sites, whereas D12, D13 and D16 were not (Figure 6G). Consistently, EXO1-D16 expressing cells displayed reduced HR (Figure 6H) and RAD51 foci formation (Figure 6I), compared to WT EXO1. Conversely, the decreased EXO1 recruitment to microirradiated sites in MSH2 depleted cells was rescued by transfection with siRNA-resistant WT MSH2, but not by the EXO1-interaction defective D9 mutant (Supplementary Figure S5B), suggesting that EXO1 recruitment to DSB requires MSH2. Since the MSH3 binding domain of EXO1 is well-characterized (38), we tested the recruitment of EXO1 to microirradiated sites using the MSH3 binding defective mutant, GFP-EXO1 D2, and the EXO1 D2 was not recruited to the DSB (Supplementary Figure S5C-D), suggesting that MSH3 is involved in EXO1 recruitment to the DSB. Taken together, EXO1 recruitment to DSB requires the MSH2-MSH3 complex, which in turn requires SMARCAD1.

To exclude the possibility that RNAi depletion alters the cell cycle profile to affect HR activity, we monitored the cell cycle profiles by FACS analysis. Depletion of MSH2, MSH3, SMARCAD1, or EXO1 did not significantly change the cell cycle profile (Supplementary Figure S5E).

### MSH2-MSH6 does not affect end resection

To investigate the effect of MSH2-MSH6, we purified human MSH2-MSH6 and performed biochemical assays in the same manner as for MSH2-MSH3. We confirmed that purified MSH2-MSH6 preferentially binds to a single-mismatch (G/T) and flap DNA, regardless of its competitors (Supplementary Figure S6A, B). We then tested whether SMARCAD1 could recruit MSH2-MSH6 to homoduplex DNA, single-mismatch DNA, and flap DNA (Supplementary Figure S6C). As shown in Figure S6C, MSH2-MSH6 bound to homoduplexes, single-mismatch DNA, and flap DNA at lower concentrations in the presence of SMARCAD1 compared to MSH2-MSH6 binding to each DNA substrate without SMARCAD1, suggesting that SMARCAD1 facilitates the binding of MSH2-MSH6 to DNA. Since SMARCAD1 affects mismatch repair through its interaction with MSH2-MSH3 and MSH2-MSH6 (33,37), both MSH2-MSH3 and MSH2-MSH6 proteins are recruited to DNA in *in vitro* experiments. Next, we tested if MSH2-MSH6 recruits EXO1. MSH2-MSH6 facilitated the recruitment of both WT and Mut EXO1-D173A proteins to a 40 bp single-mismatch DNA, regardless of competitors (Supplementary Figure S6D-E). The binding of WT EXO1 was enhanced more by MSH2-MSH6 than by Mut EXO1-D173A. Finally, we tested whether MSH2-MSH6 enhanced the exonuclease activity of EXO1 in flap DNA (Supplementary Figure S6F, G). When the MSH2-MSH6 concentration was fixed and EXO1 was titrated, the EXO1 nuclease activity was slightly increased. When MSH2-MSH6 was titrated at a fixed EXO1 concentration, no dramatic change in EXO1 nuclease activity was observed, indicating that MSH2-MSH6 slightly enhanced EXO1 nuclease activity. We suspect that the slight effect of MSH2-MSH6 on EXO1 nuclease activity is due to the structure of the flap DNA. A prior study reported that MSH2-MSH6 preferentially binds to +12 or +14 bp palindromic insertions *in vitro*, but does not repair them *in vivo* (62).

### MSH2 inhibits POLθ-mediated end-joining

**Figure 7.**
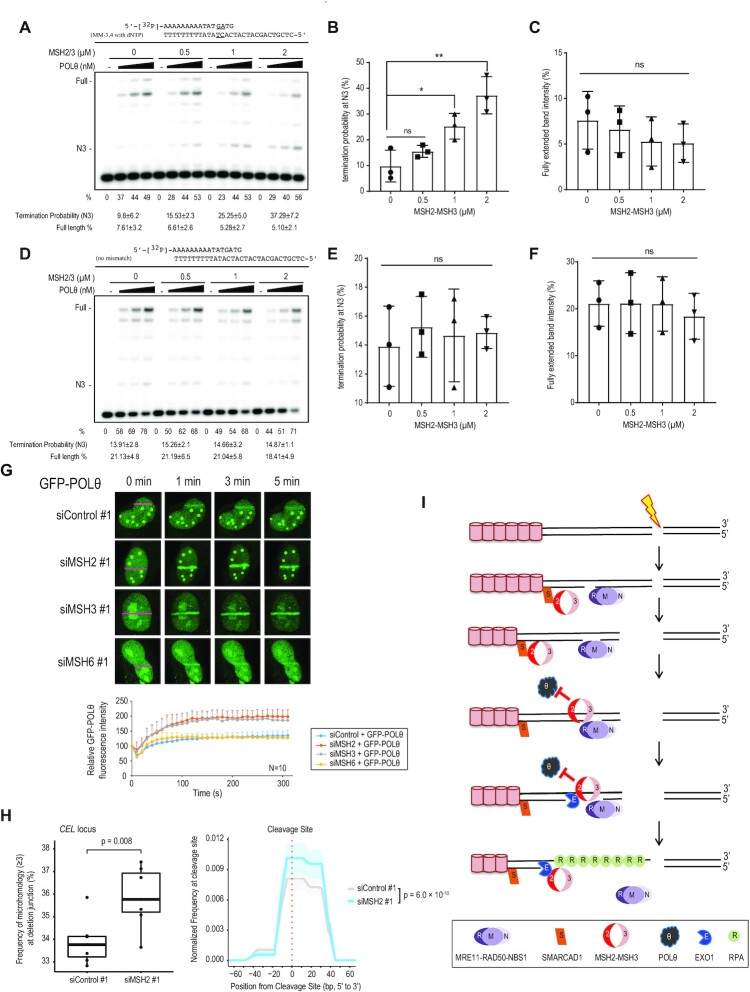
MSH2-MSH3 inhibits POLθ extension from a mismatched primer. Increasing amounts of POLθ (0.3, 0.6, and 1.3 nM) were incubated in the presence of the indicated amounts of MSH2-MSH3 and 5′-^32^P-labeled primer templates, shown at the top of the gel, at 37°C for 10 min. The first lane (−) contained no enzyme. (**A**) Percentage (%) of the product extending from the primer is shown below each lane. Two mismatched base pairs were placed at the third and fourth bp from the primer-template junction. (**B**) The termination probability at position N3 is defined as the band density at N3 divided by the intensity of ≥N3. (**C**) The quantity of full-length extension products was calculated as the fully extended band density divided by the intensity ≥ N0 (primer position). MSH2-MSH3 on non-mismatched substrates did not disturb POLθ extension (**D–F**). Data are presented as mean ± standard deviation (*n* = 3). *P*-values were calculated using two-tailed Student's *t*-test. (**G**) U2OS cells were transfected with control, MSH2, MSH3, or MSH6 siRNAs. After 24 h, cells were transfected with GFP-POLθ. Data are presented as mean + standard deviation (*n* = 10). (**H**) Mutation signatures at the CEL locus upon CRISPR-Cas9-induced DSB were compared those in control and MSH2 knockdown HEK293T cells. Boxplot showing the frequency of deletion mutations harboring microhomology longer than two nucleotides at the DNA junction out of the total deletion mutations induced by CRISPR-Cas9 targeting the CEL locus in control and MSH2 knockdown HEK293T cells. *P*-values were calculated using an unpaired two-tailed *t*-test (*n* = 7) (left). DNA deletion spectrum associated with microhomology longer than four nucleotides induced by CRISPR-Cas9 targeting the CEL locus in control and MSH2 knockdown HEK293T cells. *P*-values were calculated using a paired two-tailed *t*-test (*n* = 7) (right panel). (**I**) A model of how MSH2-MSH3 acts in the early stages of HR. Processing of only one end of the DSB is shown in the figure.

Heteroduplex DNAs are rejected by mismatch repair proteins during HR and SSA (63). DNA polymerase θ (POLθ)-mediated end-joining (TMEJ) uses the pairing of short homologous sequences (2–6 bp microhomology) of resected ssDNA to repair DSBs at the cost of generating small deletions. We hypothesized that MSH2-MSH3 might act on resected DNA to prevent POLθ recruitment, allowing for further DNA end resection and facilitating error-free HR. Such a mechanism would require POLθ to prime mismatched heteroduplex DNA. We did not observe any drastic effect on TMEJ after MSH2 knockdown (Figure 1B). However, this could be due to the sensitivity of the assay. We decided to study the relationship between POLθ and MSH2-MSH3 more directly. We tested whether POLθ could extend primers carrying a 2 bp mismatch at an internal or terminal position (Supplementary Figure S7). In a control experiment, the polymerase fragment of POLθ-catalyzed template-dependent DNA synthesis from a perfectly annealed primer pair was similar to the exonuclease-deficient *Escherichia coli* pol I Klenow Fragment (Kf exo-), another A-family DNA polymerase that served as a control (Supplementary Figure S7A) (45,64). Importantly, annealed primer pairs, which was carrying 2 bp mismatches that located 1–2 bp or 3–4 bp upstream from the 3′ primer end (MM-1, 2 and MM-3, 4), could be much more efficiently extended by POLθ than by Kf exo- (Supplementary Figures S7B, C). In other words, such extension by POLθ was particularly strong when the mismatch occurs at the primer junction (Supplementary Figure S7B) or 2 bases in the duplex (Supplementary Figure S7C). Having shown that POLθ can extend DNA synthesis using mismatched primers, we chose the MM-3, 4 substrate (Supplementary Figure S7C) to test the effect of MSH2-MSH3 on POLθ activity. POLθ activity was inhibited by MSH2-MSH3 only in the presence of a 2 bp mismatch in the double-stranded primer (Figures 7A-C), but not in the perfectly matched primers (Figures 7D-F). The termination probabilities at the N3 position were significantly increased in the presence of MSH2-MSH3 when 2 bp-mismatched substrates were used (Figures 7A-B). Consistently, the amount of fully extended products was reduced with the 2 bp-mismatched substrates (Figure 7C), but not with the no-mismatch substrates (Figures 7D-F). MSH2-MSH3 with MSH2 G674A inhibited POLθ activity more strongly than WT (Supplementary Figures S7E-G). MSH2 G674A is defective in ATP binding-induced dissociation from mismatched DNA substrates (Supplementary Figure S2E) (65). Our results indicate that MSH2-MSH3 can inhibit POLθ activity when primed with mismatched DNA substrates. To substantiate the importance of MSH2-MSH3 in preventing POLθ binding, we assessed whether POLθ recruitment to DNA damage sites is affected by MSH2, MSH3, or MSH6 depletion. POLθ recruitment was strongly increased upon MSH2 or MSH3 depletion, but not upon MSH6 depletion (Figure 7G), suggesting that MSH2-MSH3 counteracts POLθ binding to DSBs. MSH2-MSH6 also increased the termination probabilities at the N3 position when the 2 bp-mismatched substrates were used (Supplementary Figures S7H-M). However, the increased termination probability in MSH2-MSH6 (2-fold increase in 7.6 μM MSH2-MSH6) was much lower than that in MSH2-MSH3 (3.5-fold increase in 2 μM MSH2-MSH3). Therefore, the inhibition of POLθ by MSH2-MSH3 was appreciably better than that of MSH2-MSH6 *in vitro*. These results are consistent with what we observed in the microirradiation experiments, in which POLθ recruitment was only inhibited by MSH2-MSH3, not by MSH2-MSH6. We noticed that POLθ did not directly bind to MSH2, MSH3, or MSH6 proteins in *in vivo* and *in vitro* immunoprecipitation experiments (Supplementary Figures S7N, O), suggesting that enhanced POLθ recruitment to DSBs in MSH2 or MSH3 depleted cells did not depend on the physical interactions between POLθ and MSH2-MSH3. Mutation signatures in the genome can predict how DNA damage is repaired via different DNA repair pathways. The error-prone TMEJ-dependent repair often results in small deletion/insertion with microhomology signature at the breakpoint junction (66). We addressed this directly by cleaving the *CEL* locus in the genome using CRISPR-Cas9 in HeLa cells. Repaired loci were determined by targeted deep sequencing. MSH2 knockdown increased the frequency of deletions with microhomology at the breakpoint junctions (Figure 7H). Collectively, our data suggest that MSH2-MSH3 is recruited to DSBs by SMARCAD1, where it inhibits TMEJ and promotes DNA end resection by recruiting EXO1 to facilitate error-free HR.

## DISCUSSION

In the present study, we show that the MSH2-MSH3 heterodimer promotes error-free HR for DSB repair via two complementary mechanisms (Figure 7I). MSH2-MSH3 is recruited to the DSB after the initial stages of DNA end resection by SMARCAD1. SMARCAD1 and MSH2-MSH3 dependent EXO1 recruitment promotes further resection of HR. Simultaneously, the MSH2-MSH3 complex also inhibits POLθ priming and extension from mismatched DNA to prevent mutagenic TMEJ.

Based on their ability to recognize mismatched DNA sequences, MSH2-MSH3 has been implicated in the later postsynaptic stage of the HR, rejecting invading strands with imperfectly matched template DNA (26–28). MSH2-MSH3 may also inhibit hairpin structures formed during DNA end resection. MSH2-MSH3 facilitates full ATR-dependent checkpoint activation (25).

Here, we show that MSH2-MSH3 has a more direct role in HR by facilitating DNA end resection. The sequential recruitment of SMARCAD1, MSH2-MSH3, and EXO1 observed in the present study, together with the requirement of these proteins for RAD51 loading and proper HR, clearly demonstrated that MSH2-MSH3 plays an active role in the early stages of HR. In addition to inhibiting TMEJ by rejecting POLθ, MSH2-MSH3 facilitates EXO1 recruitment and long-range DNA end resection, thus funneling pathway choice towards error-free HR. HR primarily occurs in the S and G2 phases of the cell cycle. These processes are available for actively proliferating cells.

In addition to facilitating EXO1 recruitment, how could MSH2-MSH3 further aid EXO1 in DNA end resection? When MSH2-MSH3 binds to loop structures, the DNA bound by MSH2-MSH3 is bent for proper recognition by downstream proteins (67). Thus, it is possible that MSH2-MSH3 recruited to DSB sites could bend DNA to provide better access of EXO1 to DNA. MRE11-RAD50-NBS1 generates a nick and degrades ssDNA with 3′ to 5′ polarity (68). Small single-stranded gaps structurally resemble small loop structures, with the ssDNA stretch being extruded. MSH2-MSH3 recognizes such a structure (48) and bends this small-gapped DNA to provide an entry platform for EXO1 to generate long ssDNA. The recent report showing no effect of MSH3 on the formation of RPA foci differs from our results (Figure 2B and Supplementary Figure S1E). We measured RPA accumulation by two different methods, confocal microscopy and FACS. Both approaches yielded similar results. MSH2-MSH3 and XPF/ERCC1 are known to have a critical function in 3′ nonhomologous tail removal (3′ NHTR) during HR (69). Knockdown of ERCC1 reduced the frequencies of HR and SSA, but did not affect end resection (Supplementary Figure S7P). It suggests that in addition to the initial end resection by MSH2-MSH3 observed in this study, 3′ NHTR function of MSH2-MSH3 could also affect HR.

Chromatin remodeling complexes play important roles in DSB repair (43). The budding yeast Fun30 protein, an ortholog of human SMARCAD1, is a major nucleosome remodeler that enhances Exo1 and Sgs1 dependent end resection during HR repair (30). Mammalian SMARCAD1 has been suggested to play a role in HR (43). Fun30 also functions in MMR through its interaction with MSH2 (33,37). SMARCAD1, as a chromatin remodeler, may unwind chromatin structures near DSBs to help recruit MSH2-MSH3 at the early stages of DSB processing. Given the conserved interactions between SMARCAD1 and MSH2 and between MSH2 and EXO1, these interactions are conserved throughout evolution to facilitate HR. Our results clearly support a conserved mechanism by which SMARCAD1 interacts with MSH2-MSH3 and enhances the DNA binding affinity of MSH2-MSH3 (Figure 4D, E). Additionally, MSH2-MSH3 prevents the access of POLθ, the key enzyme facilitating error-prone TMEJ, to DNA damage sites and the subsequent function of POLθ. Therefore, when MSH2 or MSH3 was depleted, POLθ recruitment to DNA damage sites was enhanced.

MSH2-MSH3 (Mutsβ) and POLθ promote CAG repeat expansion during DNA replication (70–73), which may be different from DNA DSB repair. DNA replication slippage is the major mechanism for CAG repeat expansion and can be promoted by MMR and POLθ. Our observations of more POLθ recruitment to laser stripes under MSH2-MSH3 deficient conditions suggest that MSH2-MSH3 competes with POLθ at DNA DSB sites, which could be different from CAG expansion.

We expected that Lynch syndrome patients with mutations in mismatch repair proteins would have more POLθ-mediated mutation signatures in the genome. However, we did not find significant enrichment of POLθ-mediated mutation signatures in the genome of Lynch syndrome patients. It is possible that strong MMR defect signatures could be dominant in the genome of Lynch syndrome patients compared with the POLθ-mediated mutation signatures.

Analogous to its role in MMR repair (37), MSH2-MSH3 recruits EXO1 to promote DNA end resection. Interestingly, SMARCAD1 binding domains in MSH2 are shared with EXO1 binding domains. Although we did not observe competition between SMARCAD1 and EXO1 for MSH2 binding in overexpression experiments (data not shown), it is possible that this region is critical for the handover mechanism from SMARCAD1 to EXO1 facilitated by MSH2.

From an evolutionary point of view, it makes sense that key pathway proteins, such as MSH2-MSH3 dependent recruitment of EXO1, are used by more than one repair pathway. Shared modalities may also facilitate crosstalk between pathways for more efficient and coordinated repair. In the case of HR, where a single persistent DSB might lead to lethality, crosstalk may ensure that all lesions are mended. It is likely that MSH2-MSH3 is important in preventing error-prone repair by POLθ. The collective findings provide a mechanistic explanation for how the MSH2-MSH3 complex facilitates efficient DSB repair by promoting HR via recruitment of EXO1 and by preventing error-prone TMEJ by blocking POLθ access.

## DATA AVAILABILITY

All data generated or analyzed during this study are included in this published article (and its supplementary information files). Sequencing data are available on the SRA (BioProject ID: PRJNA772898).

## Supplementary Material

gkad308_Supplemental_Files

## Notes

Present address: Yuri Seo, Chungnam National University, Daejeon, Republic of Korea.

Present address: Young-Un Park, CasCure Therapeutics, Ulsan, Republic of Korea.
