# Supplementary material for: MSH2-MSH3 promotes DNA end resection during homologous recombination and blocks polymerase theta-mediated end-joining through interaction with SMARCAD1 and EXO1
Source: Nucleic Acids Res. 2023 May 4;51(11):5584–602. doi: 10.1093/nar/gkad308 (PMC10287916; doi:10.1093/nar/gkad308)
Supplement: gkad308_Supplemental_Files [file gkad308_supplemental_files.zip › Oh et al Supp Text NAR_230317 final.docx]

**MSH2-MSH3 promotes DNA end resection during homologous recombination and blocks polymerase theta-mediated end-joining through interaction with SMARCAD1 and EXO1**

Supplementary Information

Jung-Min Oh^1,2^*^+^, Yujin Kang^3^*, Jumi Park^3^, Yubin Sung^1^, Dayoung Kim^4^, Yuri Seo^1,5^, Eun A Lee^1^, Jae Sun Ra^1^, Enkhzul Amarsanaa^1,3^, Young-Un Park^1,6^, Seon Young Lee^1^, Jung Me Hwang^1^, Hongtae Kim^1,3^, Orlando Schärer^1,3^, Seung Woo Cho^1,4^, Changwook Lee^3^, Kei-ichi Takata^1,3^, Ja Yil Lee^1,3+^ and Kyungjae Myung^1,4+^

1. Center for Genomic Integrity, Institute for Basic Science (IBS), Ulsan 44919, Republic of Republic of Korea.

2. Department of Oral Biochemistry, Dental and Life Science Institute, School of Dentistry, Pusan National University, Yangsan 50612, Republic of Korea

3. Department of Biological Sciences, Ulsan National Institute of Science and Technology, Ulsan 44919, Republic of Korea

4. Department of Biomedical Engineering, Ulsan National Institute of Science and Technology, Ulsan 44919, Republic of Korea

5. Current address; Chungnam National University, Daejeon, Republic of Korea

6. Current address; CasCure Therapeutics, Ulsan, Republic of Korea

*Equal contributions

+corresponding authors

Email: jminoh@pusan.ac.kr (Jung-Min Oh), [biojayil@unist.ac.kr](mailto:biojayil@unist.ac.kr) (Ja Yil Lee) or [kmyung@ibs.re.kr](mailto:kmyung@ibs.re.kr) (Kyungjae Myung)

**Supplementary Figure Legends**

Supplementary Figure 1. MSH2 Depletion does not affect MRE11 Recruitment

(A) U2OS cells were transfected with each siRNA and incubated for 48 h. Each protein expression was analyzed by western blot. (B) Cells were transfected with the indicated siRNAs and the efficiency of HR was measured. Data are presented as mean ± standard deviation (n=3, independent cell culture). P-values were calculated by two-tailed Student's t-test. (C) After 24 h of transfection of control, MSH2, MSH3 or MSH6 siRNA, dimethyl sulfoxide (DMSO) or the indicated concentrations of baicalein were treated for 24 h and the efficiency of HR was measured. (D) HEK293T cells were transfected with control#1 or MSH6#1 siRNA and incubated for 48 h. For endogenous immunoprecipitation, cell extracts were incubated with anti-IgG or anti-MSH3 antibody. (E) Each siRNA was transfected to U2OS cells. After 2 days incubation, cells were treated with 5 μM of CPT or DMSO for 1 h. RPA foci in the nucleus were counted using confocal microscopy. Data are presented as mean ± standard deviation (n=3, independent cell culture). P-values were calculated by two-tailed Student's t-test. (F) Schematic diagram displaying the principle of end resection assay.

Supplementary Figure 2. EXO1 interacts with MSH2.

(A) Diagram of EXO1 WT and EXO1 deletion mutants. HEK293T cells were transfected with myc-MSH2 WT and GFP-EXO1 or GFP-EXO1 deletion mutants. (B) Diagram of MSH2 WT and MSH2 deletion mutants. HEK293T cells were co-transfected with myc-EXO1 WT and GFP-MSH2 or GFP-MSH2 deletion mutants. (C) HEK293T cells were co-transfected with PKC-δ-MSH2 and GFP-EXO1 WT or GFP-EXO D13. Cells were treated with 1 μM PMA for 5 min, then cell images were acquired by confocal microscopy. (D) Coomassie Brilliant Blue stained 8% Tris-Glycine SDS-PAGE gels of purified proteins used in this study. SMARCAD1, human EXO1 (wild-type (WT EXO1) and nuclease mutant (Mut EXO1-D173A)), MSH2-MSH3 (wild-type and G674A mutant), MSH2-MSH6, and POLθ were purified. (E) DNA binding analyses for MSH2-MSH3 (top) or MSH2 (G674A)-MSH3 (middle) with 40 bp homoduplex (left), +8-loop DNA (center), or 58 bp flap DNA (right) in the presence of competitors, and MSH2 (G674A)-MSH3 with 58 bp flap in the absence of competitor (bottom) at different concentrations (0, 10, 20, 40, 80, 100, 150, 200, 300, and 400 nM). (F) Exonuclease activity analysis for WT EXO1 and Mut EXO1-D173A at different concentrations (0, 10, 15, 20, 25, 30, and 40 nM). 40-bp blunt end DNA (left) or 40-bp DNA with 4-nt 3'-overhang (right) labeled with Cy5 was used as DNA substrate. DNA containing ssDNA overhang was degraded due to the digestion only by WT EXO1 in a dose-dependent manner, whereas Mut EXO1-D173A did not degrade any DNA substrate. (G) EMSA for EXO1. Duplex (left) or +8-loop DNA (right) was reacted with WT EXO1 or Mut EXO1-D173A at different concentrations (0, 10, 15, 20, 25, 30, 40, and 80 nM) in the presence of competitors. (H) Nuclease activity of WT EXO1 and Mut EXO1-D173A in the presence or absence of MSH2-MSH3 without ATP. 40 nM of 90-bp flap DNA was reacted with WT EXO1 or Mut EXO1-D173A at different concentrations (0, 5, 10, 20, 25, 30, and 40 nM) in the presence (top) or absence (bottom) of 300 nM MSH2-MSH3. Quantification of DNA degradation by EXO1 from gel images is shown below the gel images. Error bars were obtained from standard deviation in triplicate.

Supplementary Figure 3. SMARCAD1 interacts with MSH2, but not EXO1.

(A) Diagram of MSH2 WT and MSH2 deletion mutants. HEK293T cells were co-transfected with myc-SMARCAD1 WT and GFP-MSH2 or GFP-MSH2 deletion mutants. (B) HEK293T cells were co-transfected with myc-MSH3 WT and GFP-MSH2 or GFP-MSH2 deletion mutants. (C) HEK293T cell extracts were incubated with anti-IgG, anti-MSH2, anti-SMARCAD1 or anti-EXO1 antibody. (D) HEK293T cells were co-transfected with PKC-δ-MSH2 and GFP-SMARCAD1 WT or GFP-SMARCAD1 D1. (E) DNA binding analyses for SMARCAD1 with +8-loop DNA (top left) and homoduplex DNA (top center) at different concentrations (0, 50, 100, 200, 400, 800, 1000, 2000, 4000, and 8000 nM). DNA binding analysis for SMARCAD1 with 58-bp flap DNA (bottom left) at different concentrations (0, 400, 800, 1000, 2000, 4000, 8000, 16000 and 32000 nM). DNA binding analysis for MSH2-MSH3 with 58-bp flap DNA (bottom right) at different concentrations (0, 10, 20, 40, 80, 100, 150, 200, 300, and 400 nM). EMSA for SMARCAD1 and MSH2-MSH3 with a homoduplex DNA (top right). 4 μM of SMARCAD1 was incubated with a homoduplex, and then titrated with MSH2-MSH3 (0, 10, 20, 40, 80, 100, 150, 200, 300, and 400 nM).

Supplementary Figure 4. MRE11 recruitment to DSB sites is not changed by SMARCAD1 and MSH2.

(A) U2OS cells were transfected with control or MSH6 siRNA. After 24 h, cells were transfected with GFP-EXO1. The graph data of control siRNA was adopted from Figure 5C. Data are presented as mean + standard deviation (n=10). (B) HeLa cells were transfected with control, SMARCAD1, MSH2 or EXO1 siRNA. After 24 h, cells were transfected with GFP-EXO1 or GFP-MSH2. (C) U2OS cells were transfected with control, SMARCAD1, or MSH2 siRNA. After 24 h, cells were transfected with mNeon-MRE11. (D) U2OS cells were transfected with GFP-MSH2 and incubated with 10 μM of BrdU for 24 h. Transfected cells were incubated with RAD51 inhibitor B02 for the last 4 h before microirradiation (left). The effect of B02 was confirmed by HR assay. For the Rad51 siRNA experiment (right), U2OS cells were transfected with control or Rad51 siRNA. Data are presented as mean + standard deviation (n=10). (E) MLH1 knockdown, which was confirmed by western blot, did not affect HR, end resection, and GFP-EXO1 recruitment to microirradiation-induced DSB sites. End resection was measured by RPA accumulation. Data are represented as mean + standard deviation (n=10).

Supplementary Figure 5. EXO1 recruitment to DSB sites depends on MSH2

(A) Nuclear localization signal (NLS) sequences were attached to each GFP-MSH2 mutant from D6 to D9. U2OS cells were transfected with each mutant and GFP-MSH2 recruitment to the laser stripe was monitored by confocal microscopy. (B) U2OS cells were transfected with control or MSH2 siRNA. After 24 h, cells were co-transfected with GFP-EXO1 WT and mCherry-MSH2 WT or mCherry-MSH2 D9 mutant. (C) HEK293T cells were co-transfected with GFP-EXO1 and myc-MSH2, myc-MSH3, or myc-MSH6. HEK293T cell extracts were incubated with anti-myc antibody for IP. Diagram of EXO1 WT and EXO1 deletion mutants. HEK293T cells were transfected with myc-MSH3 WT and GFP-EXO1 or GFP-EXO1 deletion mutants. (D) U2OS cells were transfected with GFP-EXO1 WT or GFP-EXO1 D2 mutant. Data are presented as mean + standard deviation (n=10). (E) U2OS cells were transfected with control, SMARCAD1, MSH2, MSH3 or EXO1 siRNA. After 48 h, cell cycle was measure by FACS analysis.

Supplementary Figure 6. MSH2-MSH6 does not affect end resection.

(A) DNA binding analyses for MSH2-MSH6 in the absence of competitor with 40-bp homoduplex (left), single-mismatch (G/T) DNA (middle), and 58-nt flap DNA (right) at different concentrations (0, 10, 20, 40, 50, 60, 70, 80, 90, 100, 125, and 150 nM). Both 40-bp homoduplex and single-mismatch DNA have Cy5 at 5' end of one strand. MSH2-MSH6 shows preferential binding to single-mismatch DNA. (B) DNA binding analyses for MSH2-MSH6 with 40-bp homoduplex (left), single-mismatch (G/T) DNA (middle), and 58-bp flap DNA (right) in the presence of competitor (50 nM of unlabeled 40-bp homoduplex). All experimental conditions except the addition of competitor are identical to (A). (C) EMSA for SMARCAD1 and MSH2-MSH6. 4 μM SMARCAD1 was bound to 40-bp homoduplex DNA (top left), single-mismatch DNA (top right), or 58-nt flap DNA (bottom left). MSH2-MSH6 was titrated (0, 10, 20, 40, 50, 60, 70, 80, 90, 100, 125, and 150 nM). (D, E) EMSA for MSH2-MSH6 and EXO1. 80 nM MSH2-MSH6 was bound to single G/T mismatch DNA, and WT EXO1 or Mut EXO1-D173A was then titrated (0, 10, 15, 20, 25, 30, 40, and 80 nM) in the absence (D) or presence (E) of 50 nM unlabeled 40-bp homoduplex competitors. (F) Nuclease activity of EXO1 for 90-bp flap DNA in the presence or absence of MSH2-MSH6 at 1 mM ATP. 40 nM DNA with 90-bp flap DNA was reacted with WT EXO1 (left) or Mut EXO1-D173A (right) at different concentrations (0, 5, 10, 20, 25, 30, 40, and 60 nM) in the presence (top) or absence (bottom) of 300 nM MSH2-MSH6. Quantification is shown below the gel images. Error bars were obtained from standard deviation in triplicate. (G) Nuclease activity of EXO1 in the titration of MSH2-MSH6. 40 nM of 90-bp flap DNA was reacted with 20 nM WT EXO1 (top) or EXO1-D173A (bottom) at different concentrations (0, 10, 30, 100, 300, and 500 nM) of MSH2-MSH6. The EXO1 nuclease activity was quantified below the gel images. Error bars were obtained from standard deviation in triplicate.

Supplementary Figure 7. POLθ efficiently extends from a mismatched primer end.

Increasing amounts of POLθ (0.3, 0.6, 1.3, 0.25, 0.5, 10, 20, and 40 nM) and KF (exo-) (3.9, 7.8, 15.6, 31.3, 62.5, 125, 250, and 500 fM) were incubated with the 5′-^32^P-labeled primer templates indicated above the gel in the presence of all 4 nt at 37°C for 10 min. The first lane contains no enzymes (−). The percentage (%) of product extension from the primers is shown below each lane. (A) No-mismatch; two mismatched base pairs were placed 1 and 2 bp (B), 3 and 4 bp (C), and 5 and 6 bp (D) from the primer-template junction.

(E-G) Increasing amounts of POLθ (0.3, 0.6, 1.3 nM) were incubated in the presence of the indicated amounts of wild-type or mutant MSH2-MSH3 with the 5′-^32^P-labeled primer templates indicated above the gel in the presence of all 4 nt at 37°C for 10 min. The first lane contains no enzymes (−). The percentage (%) of product extension from the primers is shown below each lane. Two mismatched base pairs were placed 3 bp and 4 bp from the primer-template junction. (E) POLθ was incubated with wild-type MSH2-MSH3 and mismatched substrates (E), mutant MSH2-MSH3 and mismatched substrates (F), or mutant MSH2-MSH3 and non-mismatched substrates (G). (H) To check the POLθ activity on mismatched substrate in the presence of MSH2-MSH6, the percentage (%) of the product extended from the primer is shown below each lane. (I) The termination probability at position N3 is defined as the band density at N3 divided by the intensity of ≥ N3. (J) The quantity of full-length extension products was calculated as the fully extended band density divided by the intensity ≥ N0 (primer position). The effects of MSH2-MSH6 on the matched substrate were similarly measured (K-M). Data are presented as mean ± standard deviation (n=3). P-values were calculated using two-tailed Student's t-test. (N) HEK293T cells were co-transfected with GFP- POLθ, myc-MSH2, myc-MSH3, or myc-MSH6. HEK293T cell extracts were incubated with an anti-myc antibody, and a GFP antibody was used for western blotting. (O) Immunoprecipitation assays for purified POLθ and MSH2-MSH3 proteins. The indicated antibodies were used for the western blot analysis. (P) The efficiency of HR, end resection, and SSA was measured after transfection of control or ERCC1 siRNA. Data are represented as mean ± standard deviation (n=3, independent cell culture). P-values were calculated by two-tailed Student's t-test.

**Supplementary Table. List of oligomers**

| Name | Sequence |
| --- | --- |
| 40 bp  homoduplex | 5’-ACCGAATTCTGACTTGCTAGGACATCTTTGCCCACGTTGA-3’ |
|  | 5’-**Cy5**-TCAACGTGGGCAAAGATGTCCTAGCAAGTCAGAATTCGGT-3’ |
| +8-loop DNA* | 5’-ACCGAATTCTGACTTGCTAGGTGTGTGTGACATCTTTGCCCACGTTGA-3’ |
|  | 5’-**Cy5**-TCAACGTGGGCAAAGATGTCCTAGCAAGTCAGAATTCGGT-3’ |
| 40 bp  GT mismatch | 5’-ACCGAATTCTGACTTGCTAGGGCATCTTTGCCCACGTTGA-3’ |
|  | 5’-**Cy5**-TCAACGTGGGCAAAGATGTCCTAGCAAGTCAGAATTCGGT-3’ |
| 40 bp  overhang** | 5’-ACCGAATTCTGACTTGCTAGGACATCTTTGCCCACGTTGATTTT-**Cy5**-3’ |
|  | 5’-TCAACGTGGGCAAAGATGTCCTAGCAAGTCAGAATTCGGT-3’ |
| 58 bp flap DNA*** | 5’- GTGCACTCTCAGTACAATCTGCTCTGATGC**TTTTTTT** -3’ |
|  | 5’- TAAGCCAGCCCCGACACCCG **Cy5**-3’ |
|  | 5’- CGGGTGTCGGGGCTGGCTTAACTATGCGGCATCAGAGCAGATTGTACTGAGAGTGCAC -3’ |
| 90 bp flap DNA*** | 5’-ACGCATCTGTGCGGTATTTCACACC**TTTTTTTTTTTTTTTTTT**-**Cy3**-3’ |
|  | 5’-TCAGTACAATCTGCTCTGATGCCGCATAGTTAAGCCAGCCCCGACACCCG-**Cy5**-3’ |
|  | 5’-CGGGTGTCGGGGCTGGCTTAACTATGCGGCATCAGAGCAGATTGTACTGAGAGTGCACCATATGCGGTGTGAAATACC  GCACAGATGCGT-3’ |

* Red represents 8-nt bubble.

** Green represents GT mismatch.

*** Blue represents 4-nt single-stranded overhang.

**** Bold represents single-stranded flap.
