# Supplementary figures and images for: MSH2-MSH3 promotes DNA end resection during homologous recombination and blocks polymerase theta-mediated end-joining through interaction with SMARCAD1 and EXO1
Source: Nucleic Acids Res. 2023 May 4;51(11):5584–602. doi: 10.1093/nar/gkad308 (PMC10287916; doi:10.1093/nar/gkad308)

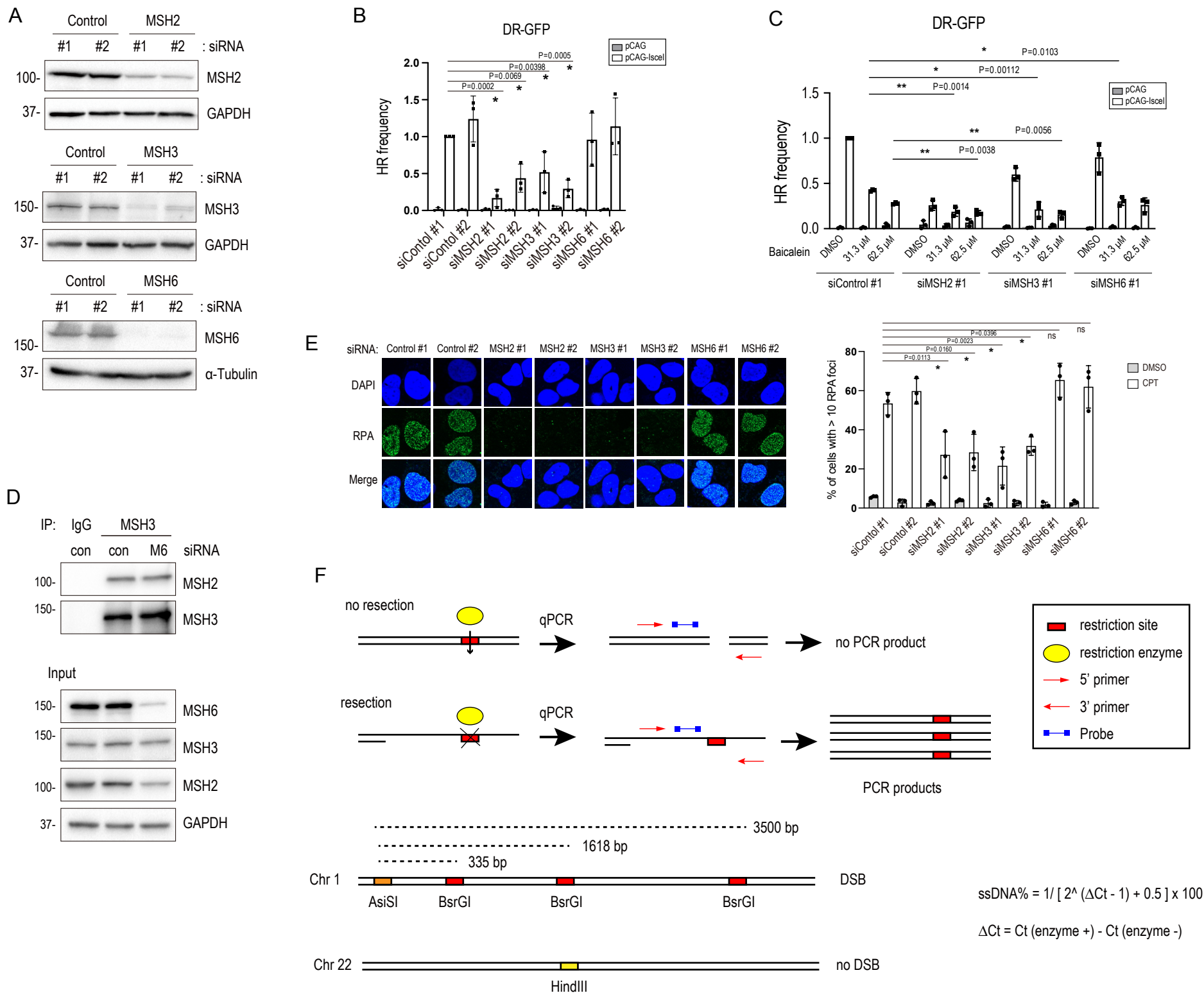

Figure S1. Oh et al.

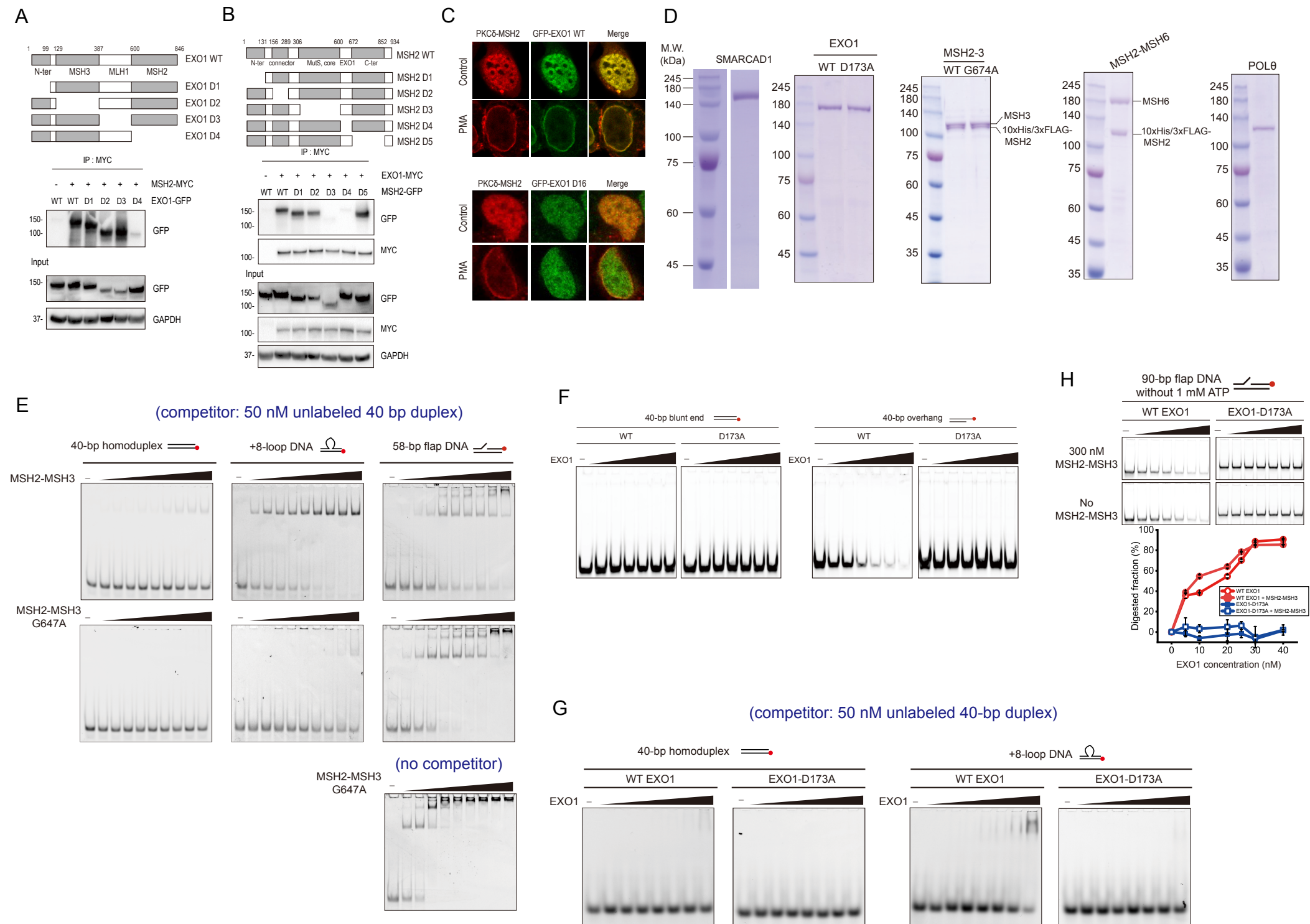

Figure S2. Oh et al.

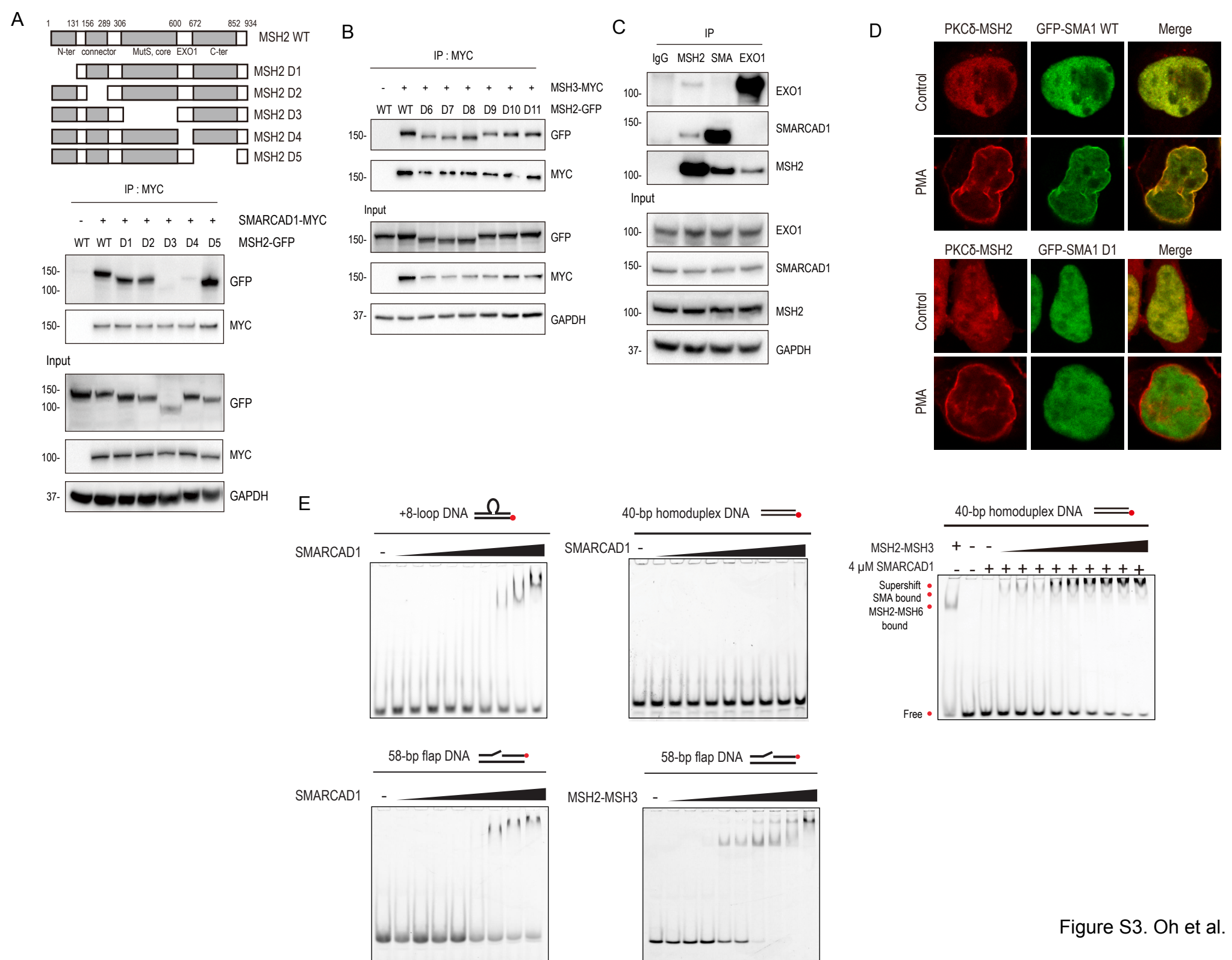

Figure S3. Oh et al.

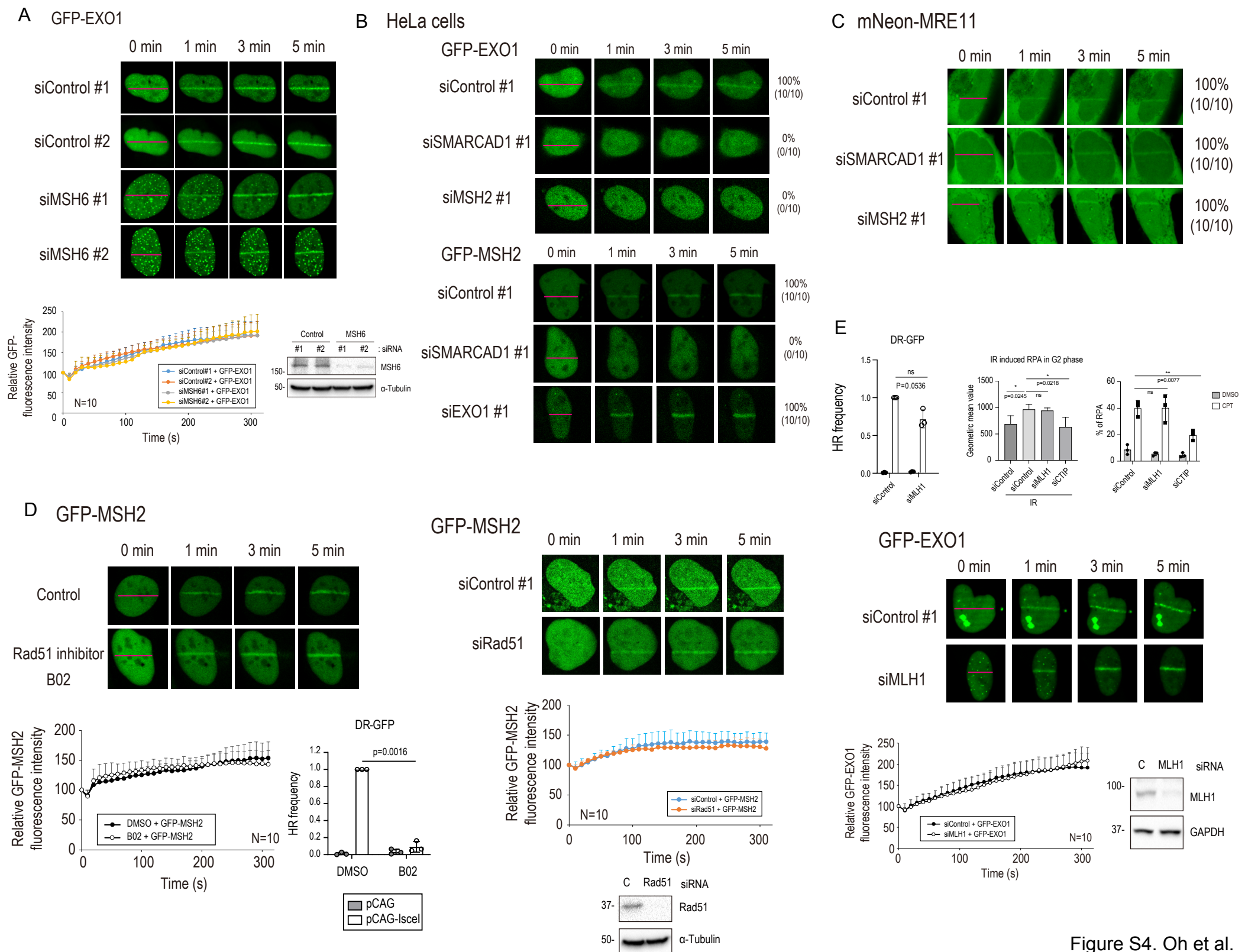

Figure S4. Oh et al.

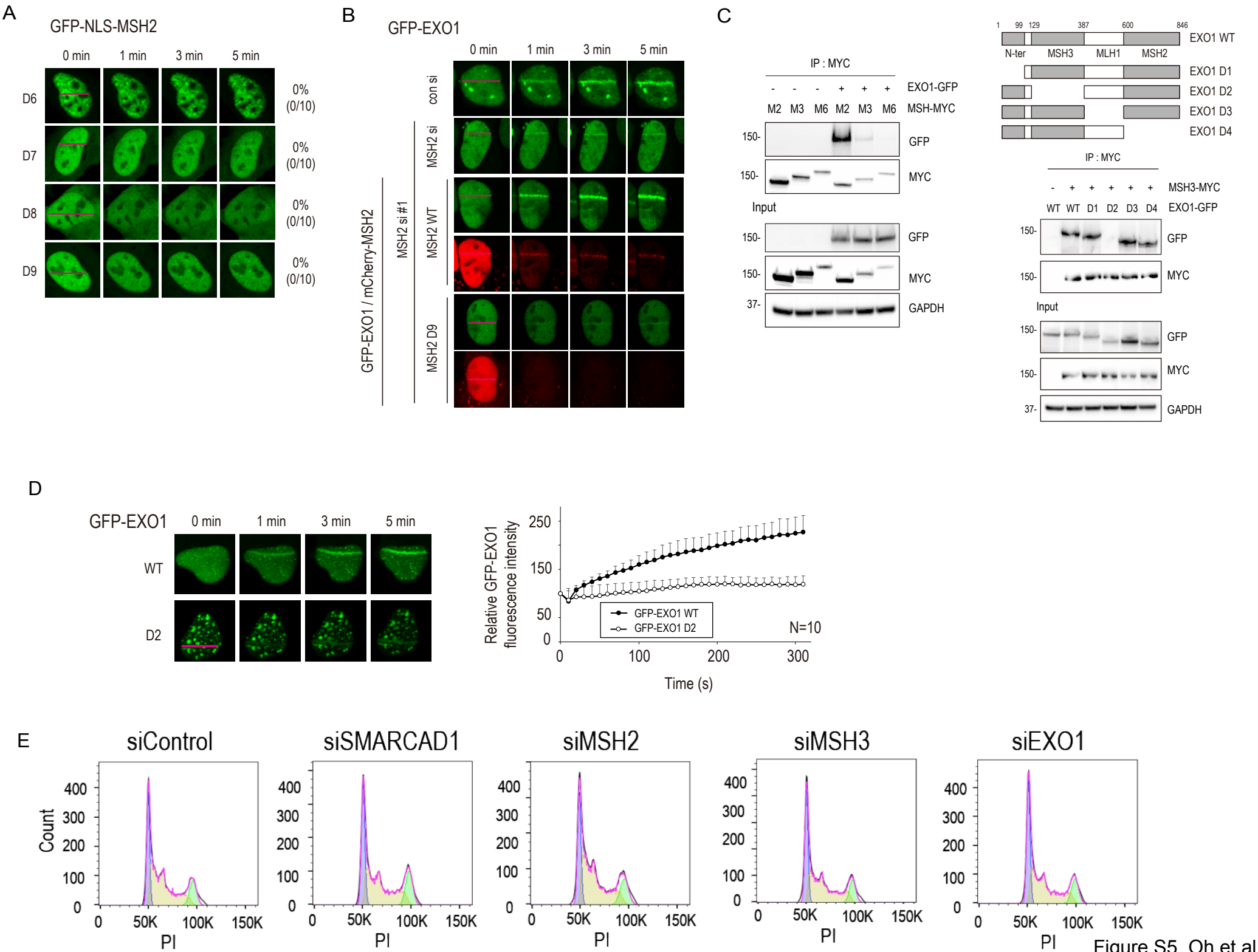

Figure S5. Oh et al.

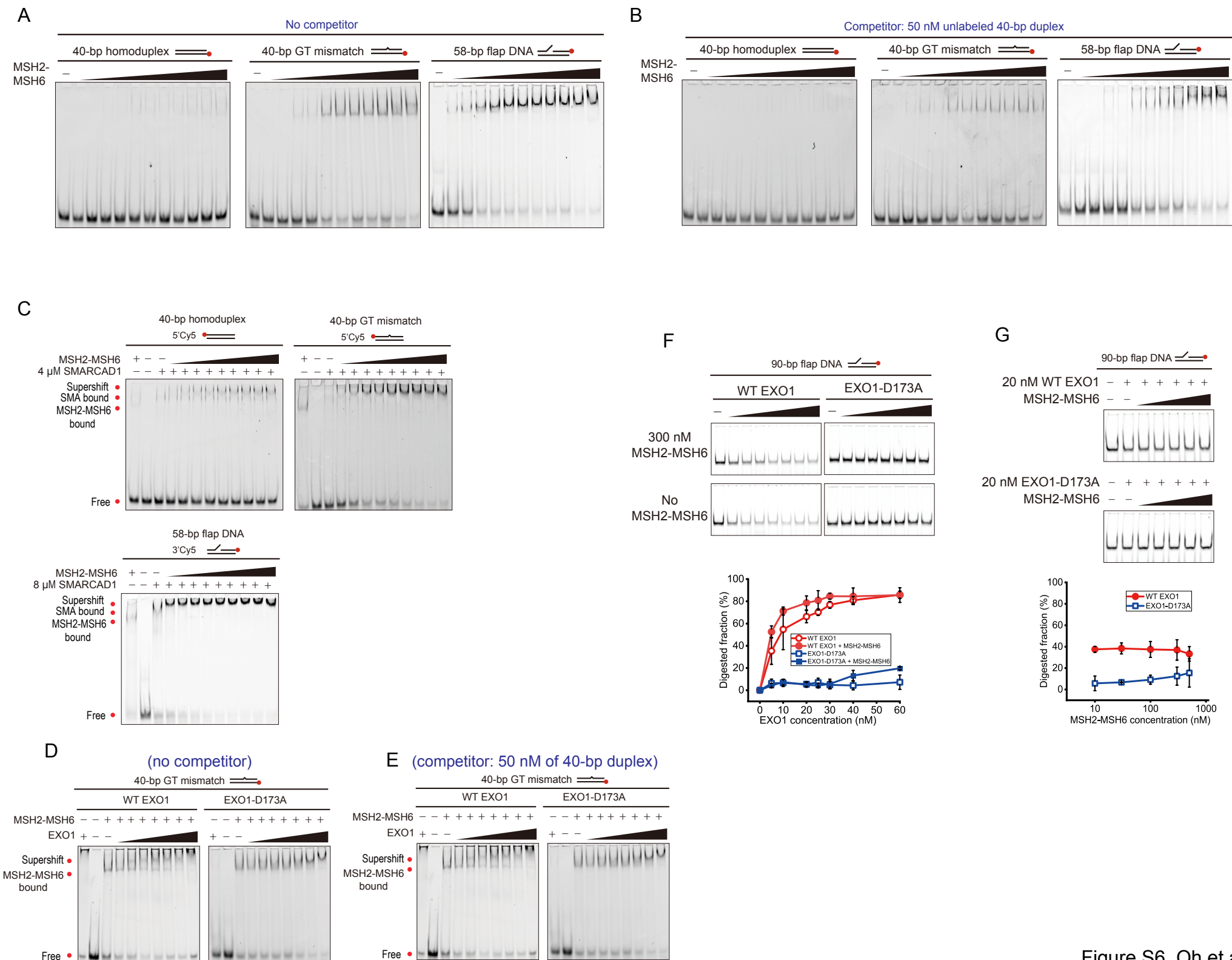

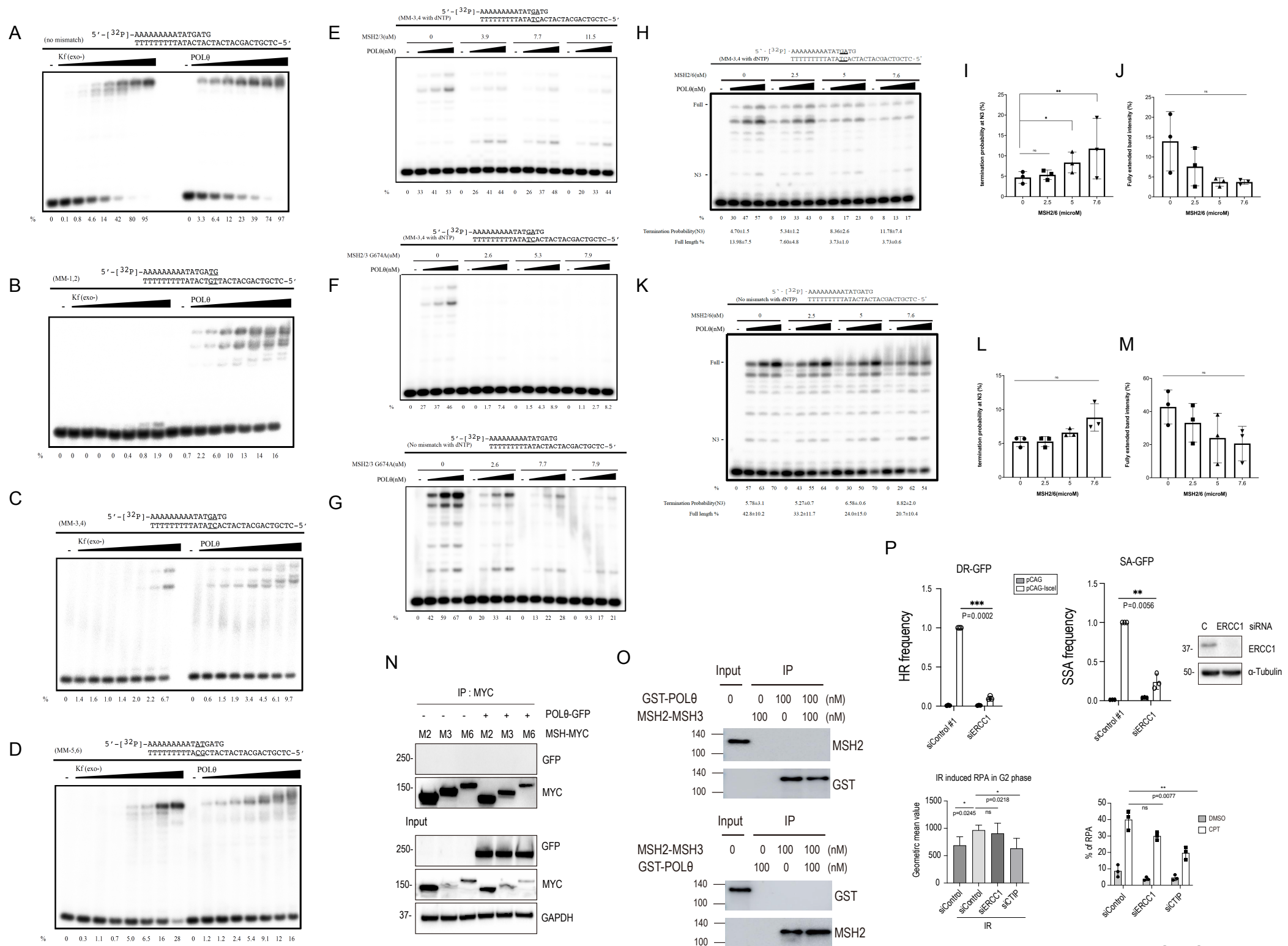

Figure S7. Oh et al.

Supplement: gkad308_Supplemental_Files [file gkad308_supplemental_files.zip › Supplementary Figures_revision final.pdf]
